# Influence of Temperature and Osmolytes on Conformational Dynamics of Alpha Synuclein

**DOI:** 10.1021/acs.jpcb.6c04859

**Published:** 2026-10-13

**Authors:** Jared Hampton, Cameron Lynch, Robert L. Rider, Carter Lantz, Zhenyu Xi, David H. Russell

**Affiliations:** Department of Chemistry, 14736 Texas A&M University, College Station, Texas 77843, United States

## Abstract

Alpha-synuclein (α-syn) is an intrinsically disordered protein (IDP) associated with amyloid formation in neurodegenerative diseases, including Parkinson’s, dementia with Lewy bodies, and multiple system atrophy. Disease progression is linked to the transition of α-syn from native to misfolded conformations, although the mechanisms underlying this process remain unclear. An effective method for investigating protein dynamics is native mass spectrometry, specifically when coupled with ion mobility-mass spectrometry (IM-MS) to examine differences in the shape and size of various analytes. In this study, variable-temperature electrospray ionization ion mobility-mass spectrometry (vT-ESI-IM-MS) was used to investigate the conformational dynamics of wild-type and A30P α-syn in the presence and absence of five osmolytes trimethylamine-*N*-oxide (TMAO), urea, guanidine, 1-dodecylguanidine (dodine), and arginine. For both α-syn variants, the abundance of extended conformers increased with temperature in the absence of osmolytes, consistent with temperature-dependent conformational shifts. This study shows osmolytes significantly alter conformational distributions and temperature-dependent responses, with dodine promoting extended conformations and guanidine producing the lowest abundance of extended states. These results demonstrate the utility of vT-ESI-IM-MS for probing IDP dynamics and highlight the influence of osmolytes on IDP conformational behavior.

## Graphical abstract

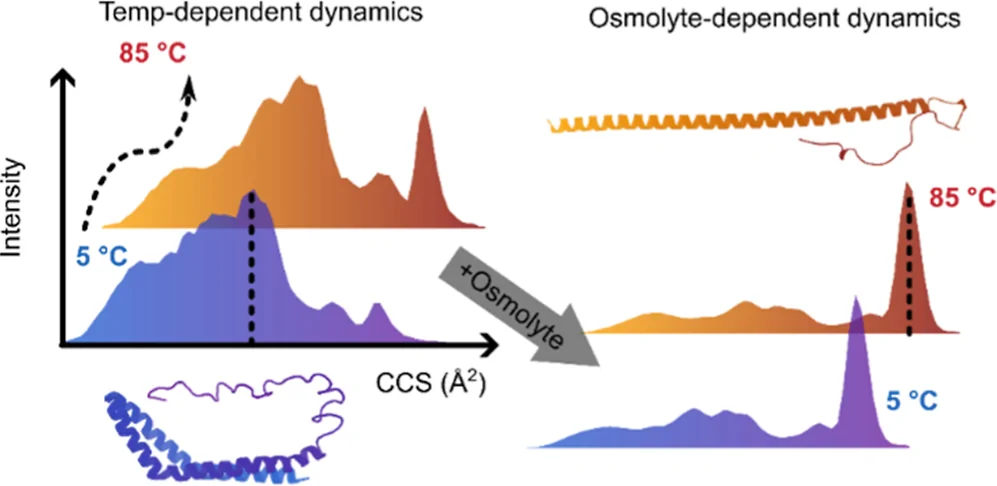

## Introduction

Alpha-synuclein (α-syn) is a 140 residue (14.4 kDa) intrinsically disordered protein (IDP) that is highly prone to amyloid formation.^1^ This protein comprises three distinct domains: an N-terminal domain (residues 1–60) that forms amphipathic α-helices upon phospholipid binding, a central non amyloidal component (NAC; residues 61–95) that drives fibrillogenesis, and a C-terminal domain (residues 96–140) involved in protein interactions and metal binding.^2–4^ Alpha-synuclein amyloidosis (synucleinopathy), is implicated in several neurodegenerative diseases, including Parkinson’s disease (PD), dementia with Lewy bodies (DLB), and multiple system atrophy (MSA).^5,6^ These conditions are associated with the formation of oligomers and insoluble α-syn fibrils arising from perturbations of the native conformational ensemble that promote aggregation-prone intermediates. Familial mutations, including A30P, E46K, and A53T, further modulate the physicochemical properties and aggregation behavior of α-syn relative to wild-type (WT).^7,8^ Although the factors responsible for the transition from native to aggregation-prone α-syn conformations remain poorly understood, osmolytes could provide a useful means of modulating protein–solvent interactions and may offer insight into the conformational dynamics that promote compact or extended α-syn states.

Osmolytes are small organic molecules that can be classified into several subclasses, including amino acids and derivatives, polyols, methylamines, and methylsulfoniums, and are known to influence protein dynamics by modulating protein hydration and stability via noncovalent interactions.^9^ Although the precise mechanisms remain unclear, evidence suggests that osmolytes alter nonpolar interactions and hydrogen-bonding networks, thereby stabilizing or destabilizing specific protein conformations.^10,11^ Dodine has been shown to destabilize α-helical structure while largely preserving β-sheet content and exhibits bifunctional behavior owing to a polar guanidinium group with a nonpolar 12 chain carbon tail, stabilizing proteins at low concentrations and destabilizing them at higher concentrations.^12,13^ Trimethylamine-*N*-oxide (TMAO) and arginine have been reported to be protein stabilizers, suppressing protein–protein interactions and inhibiting aggregation, whereas guanidine and urea are well-established protein destabilizers that promote protein unfolding.^14,15^ Although osmolyte effects on globular proteins are well characterized, their influence on IDPs remains less understood. For example, studies by Bhat et al. demonstrated that TMAO destabilizes α-casein, whereas other methylamine osmolytes do not, suggesting that osmolyte effects on IDPs may differ substantially from those observed for folded proteins.^16^ Previous spectroscopic studies of TMAO and α-syn have reported biphasic changes in protein folding, while molecular dynamics simulations have shown that urea and TMAO can alter secondary structure from compact to extended conformers.^17–19^ To investigate osmolyte-induced changes for the conformational dynamics of α-syn, native mass spectrometry coupled with ion mobility provides a powerful approach for investigating heterogeneous conformational dynamics.

Native MS (nMS) provides a powerful approach for studying proteins, protein complexes, protein–protein interactions, and ligand binding by preserving solution-phase structures via “freeze-drying” of biomolecules with electrospray ionization (ESI).^20,21^ Previous nMS studies have employed osmolytes as charge reducing agents to minimize charging on proteins and protein complexes.^22,23^ Ion mobility-mass spectrometry (IM-MS) further complements nMS by separating ions according to charge, size, and shape enabling the observation of different conformations.^24^ IM-MS has been applied to characterize the misfolding of amyloidogenic proteins, including transthyretin, amyloid beta, and α-syn.^25–28^ In addition, IM-MS has been used to investigate oligomerization of amyloid proteins and therapeutic peptides such as liraglutide.^25,29–31^ Variable-temperature nMS experiments have further enabled the study of protein thermodynamics through measurements of temperature-dependent changes in charge-state distributions and ligand binding.^32–34^ Integration of variable-temperature ESI IM-MS (vT-ESI-IM-MS) further expands these capabilities by enabling direct observation of temperature-dependent conformational dynamics. Previous studies have demonstrated the effectiveness of vT-ESI-IM-MS for characterizing temperature-induced structural changes and thermodynamics for proteins such as chymotrypsin inhibitor II (CI-2) and ubiquitin.^24,35–37^ However, although gas-phase heating IM-MS experiments have been performed, no vT-ESI-IM-MS studies have examined the effects of solution-phase heating on α-syn.^38^

Here, vT-ESI-IM-MS was used to investigate the effects of temperature and osmolytes on the conformational dynamics of WT and A30P α-syn. Differences between the variants provide insight into the role of protein–solvent interactions in α-syn dynamics, which are known to be strongly influenced by water.^39^ To further probe these interactions, five osmolytes with distinct physicochemical properties were examined: TMAO and urea, well-established protein stabilizing and destabilizing agents, respectively; guanidine, dodine (a substituted guanidine derivative), and arginine, which differ in hydrophobic and hydrophilic character. Osmolyte-induced changes in conformational distributions were used to investigate solvent-mediated effects on α-syn dynamics, which have been linked to localized structural fluctuations and hydration dynamics in IDPs.^40^ Importantly, the number of conformers contributing to the collision cross section (CCS) distributions remained consistent across the conditions examined, indicating that changes in the distributions primarily reflect shifts in the relative populations of existing conformational states rather than changes in conformational heterogeneity. Overall, this study highlights the utility of vT-ESI-IM-MS for characterizing IDP conformational dynamics and the response of IDPs to perturbations resulting from changes in hydration induced by temperature and osmolytes.

## Method

### α-Syn and Osmolyte Sample Preparation

Wild type alpha synuclein (WT α-syn) and the A30P mutant of α-syn were purchased from rPeptide (Watkinsville, Georgia) and stored as 1 mg/mL aliquots in LC–MS grade water (Fisher Scientific, Ottawa, ON). The α-syn samples were buffer exchanged into 20 mM ammonium acetate (Sigma-Aldrich, St. Louis, MO) using Bio-Spin 6 SEC columns (BioRad, Hercules, CA) and diluted to a final α-syn concentration of 10 μM for analysis. Solutions (100 μM) of the osmolytes TMAO (Cayman Chemical, Ann Arbor, MI), urea, 1-dodecylguanidine (dodine), guanidine, and arginine (all from Sigma-Aldrich, St. Louis, MO) were prepared to which the α-syn samples were subsequently added. Chemical structures of the osmolytes are shown in Scheme 1. The α-syn osmolyte samples were incubated at room temperature for 15 min prior to IM-MS analysis.

**Scheme 1:**
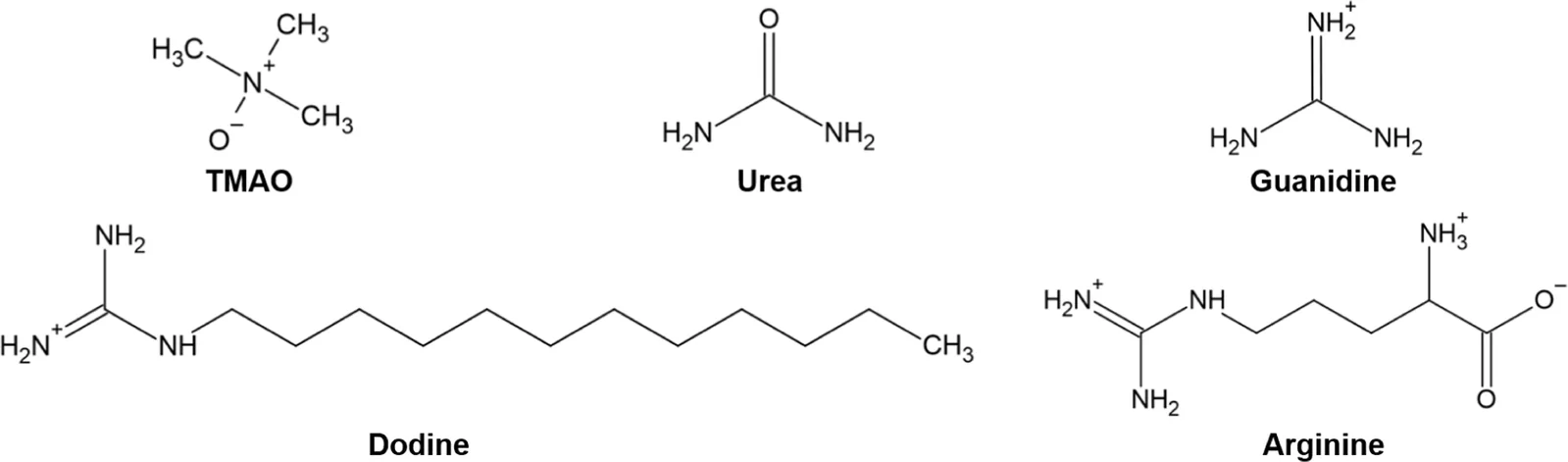
Chemical Structures of the Five Osmolytes (TMAO, Urea, Guanidine, Dodine, and Arginine) at pH 6.8 Used in This Study

### Variable Temperature IM-MS Configuration

Variable-temperature ESI IM-MS (vT-ESI-IM-MS) was employed to study the effect of temperature on the conformational dynamics of α-syn. Experiments were performed on an Agilent 6560 IM-QTOF mass spectrometer modified with a variable-temperature (vT) ESI device developed by McCabe et al.^33^ Design changes include extending the heatsink from 15 mm to 25 mm to ensure the electrospray tip was maintained at the desired temperature during analysis. The variable temperature device on the mass spectrometer is pictured in the Supporting Information (Figure S1).

### vT-ESI-IM-MS Instrument Parameters and Data Analysis

Variable-temperature (vT) ESI-IM-MS was conducted on an Agilent 6560 IM-QTOF mass spectrometer (Agilent Technologies, Santa Clara, CA) by using custom borosilicate capillaries fabricated with a P-1000 micropipette puller (Sutter Instrument, Novato, CA) and gold coated with a Leica EM ACE200 sputter coater (Leica Microsystems, Wetzlar, Germany). Triplicate measurements were obtained for each condition, with IM-MS data collected over 1.5 min in single-field ion mobility mode. Following each temperature change of the vT device, samples were equilibrated for 3 min prior to analysis to ensure the solution reached the target temperature. Instrument parameters include a spray voltage of 1650 V, gas temperature of 325 °C, a drying gas flow rate of 5 L/min, a fragmentor voltage of 250 V, and a collision cell voltage of 50 V. These instrument conditions were validated on the model protein cytochrome *c* and showed retention of native conformers that agree with past results from the McClean lab (Figure S2).^41^ Data were processed using MassHunter and plotted with Origin 2026 (OriginLab, Northhampton, MA). Drift times were converted to collision cross section (CCS) using the previously established single-field method.^42^ Average charge state was determined using UniDec.^43^ Additionally, individual fits for the 10+ and 9+ charge states were obtained using the minimum number of Gaussians to fit the distribution with a custom python script based on previous work by the Clemmer, Bleiholder, and Bush laboratories.^44–47^ An example fit and the fitting parameters are detailed in the Supporting Information (Figure S3 and Table S1).

## Results

To investigate the conformational dynamics of α-syn, WT and the A30P variant were selected because of their different aggregation propensities.^48^ ESI temperatures ranging from 5 to 85 °C were employed to evaluate the effects of solution-phase heating on α-syn conformational behavior. Temperature-dependent shifts in the abundance of compact and extended conformers were observed, likely reflecting the role of protein–water interactions in modulating α-syn dynamics as well as the internal energy of α-syn increasing with increased temperatures. Five osmolytes with known shifts in dynamics via noncovalent interactions were further examined to assess their effects on WT and A30P. The presence of osmolytes generally attenuated the temperature-dependent conformational changes and altered the relative abundances of α-syn conformer populations in distinct ways depending on the osmolyte.

### WT and A30P Have Different Conformational Dynamics Revealed by IM-MS

Native MS and IM-MS data were acquired for WT α-syn and the A30P variant (Figure 1). The intrinsically disordered nature of α-syn is evident from the conformational heterogeneity observed across all charge states in the IM-MS data. The mass spectra of both proteoforms exhibit charge states from 14+ to 6+, with the 12+ charge state being the most abundant (Figure 1A,B). Corresponding IM-MS data (Figure 1C,D) show that higher charge states adopt more extended conformations, whereas lower charge states are more compact, consistent with higher charge states being unfolded. Notably, native protein conformations have been reported to carry approximately 80% of the Rayleigh limit charge, corresponding to 7.6 charges for α-syn.^49^ Charge states exceeding 100% of the Rayleigh limit (9.5 charges) have previously been associated with unfolded conformations between native (7–6+) and unfolded (15–10+) states (Figure 1C,D). Furthermore, for charge states from 11+ to higher charge states, only extended conformations are populated. For both variants, the 10+ and 9+ charge states display the greatest conformational heterogeneity, comprising substantial populations of compact, intermediate, and extended regions. These highly dynamic charge states were therefore selected for further analysis to evaluate the effect of temperature on α-syn conformational dynamics.

**Figure 1:**
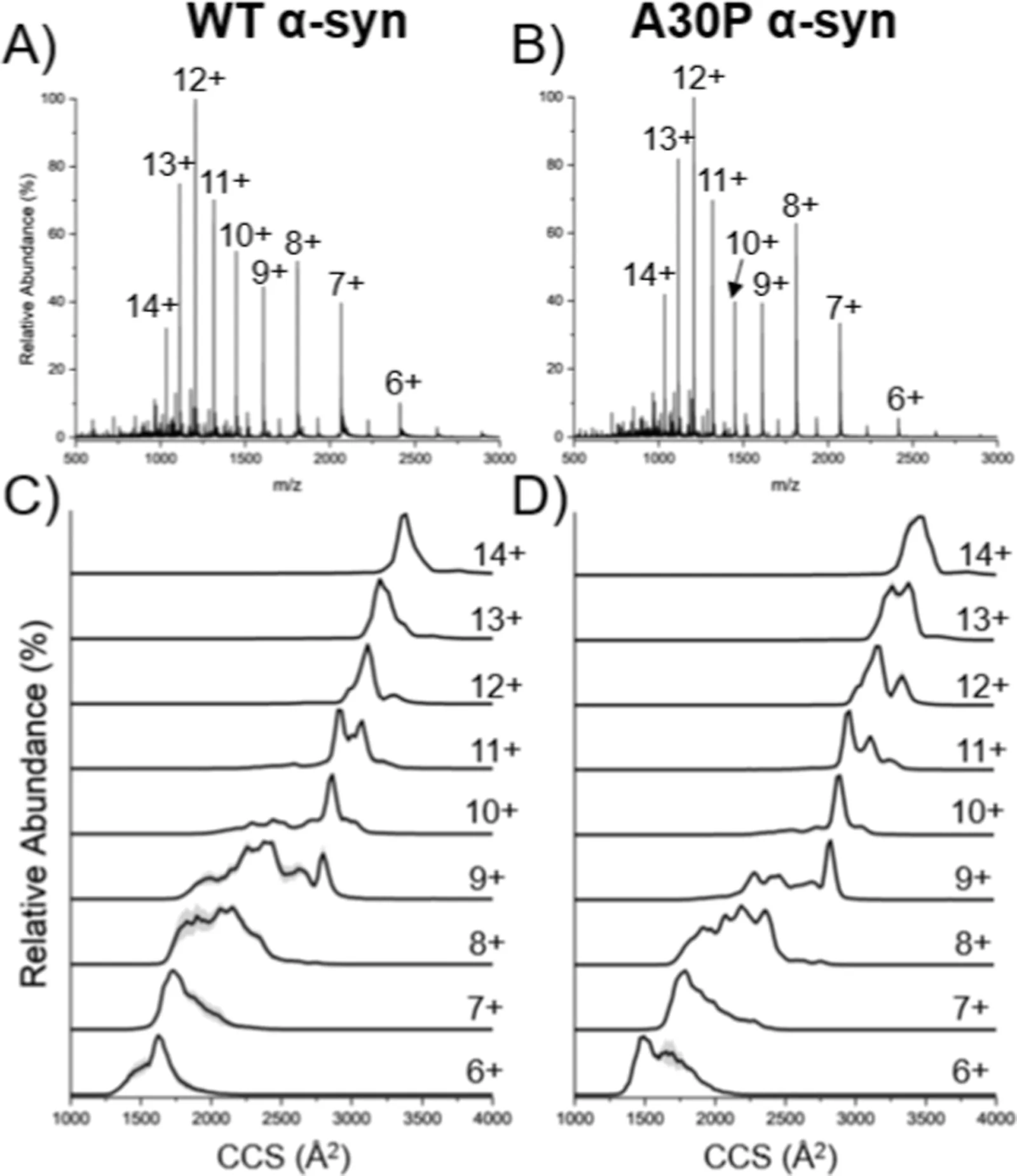
Native mass spectra and IM-MS mobiligrams of WT and A30P α-syn acquired using a commercial electrospray ionization Agilent source. Mass spectra for WT (A) and A30P (B) are shown with the corresponding IM-MS mobiligrams (C,D). Mobiligrams are displayed for charge states 6+ to 14+ and consist of *n* = 3 replicates. Low-abundance dimers are observed for both variants, along with residual tune mix ions below 1500 *m*/*z*.

### vT-ESI-IM-MS Shows Temperature-Dependent Population Shifts for WT and A30P α-syn

Variable temperature (vT) ESI-IM-MS data were acquired to examine the effects of temperature on the 10+ and 9+ charge states of WT and A30P α-syn from 5 to 85 °C (Figure 2). The mass spectra show no significant change in charge state distributions with temperature (Figure S4). Dashed lines in Figure 2 represent fits for distinct conformational populations, with 7 conformers being observed for the 10+ charge state and 8 conformers for the 9+ charge state (see Supporting Information, Figure S2). For the 10+ charge state of both variants, increasing temperature seemed to elicit a decrease in the compact conformers with an increase in the abundance of extended conformers (Figure 2A,C). A similar trend is observed for the 9+ charge state, where lower temperatures favor compact conformations with minimal extended populations, while higher temperatures increase the abundance of extended conformers (Figure 2B,D). Both charge states exhibit temperature-dependent population shifts, with higher temperatures promoting more extended conformations.^50^ This trend aligns with molecular dynamics simulations that show a change in secondary structure for α-syn in response to temperature.^17^ When comparing the two charge states, the 9+ exhibited a greater change in dynamics in response to changes in temperature and across the 2 variants compared. All charge states at the different ESI temperatures for both WT and A30P can be found in the Supporting Information where little to no changes are observed across the charge states outside those for the 10+ and 9+ charge state (Figure S5). Following the observation of temperature-dependent conformational shifts in WT and A30P α-syn, we investigated the effects of osmolytes on their conformational dynamics.

**Figure 2:**
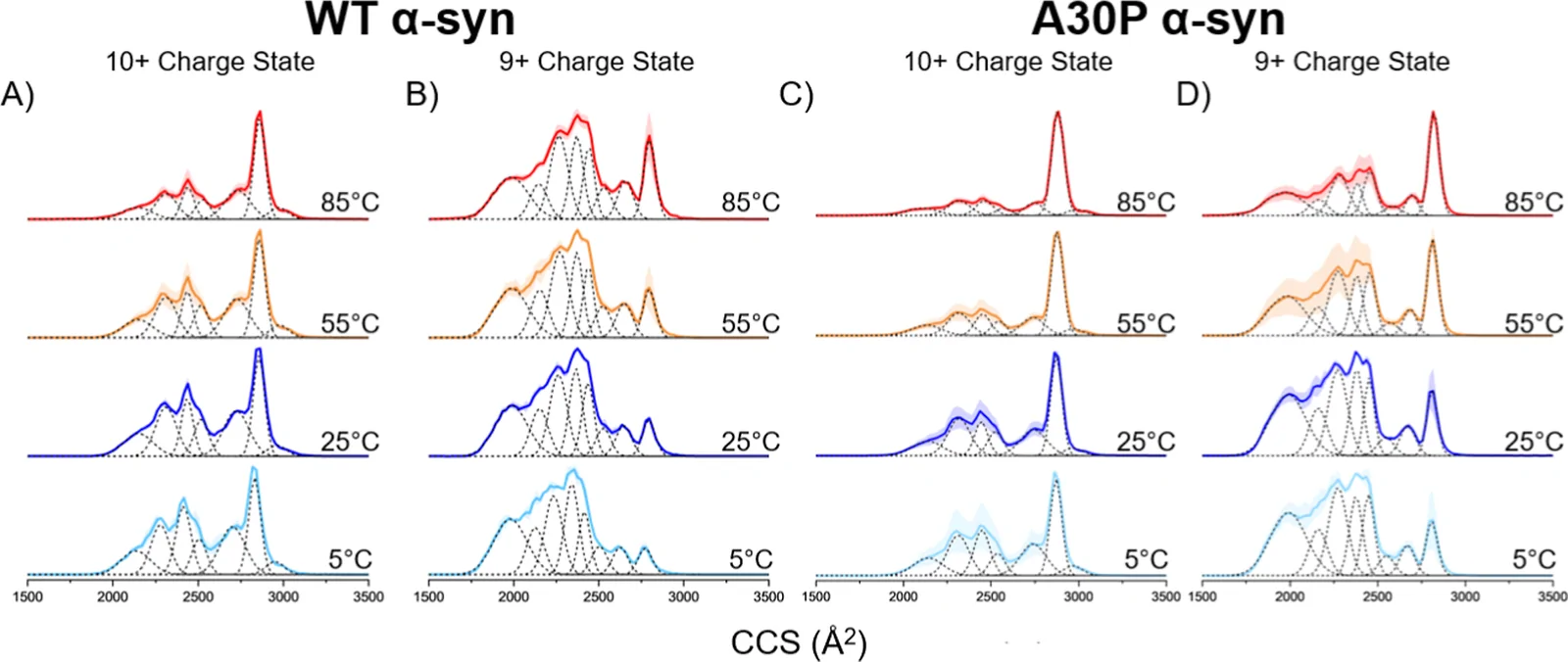
vT-ESI-IM-MS mobiligrams of WT and A30P α-syn in 20 mM AmAc for the 10+ and 9+ charge states at temperatures of 5 (light blue), 25 (blue), 55 (orange), and 85 °C (red). Mobiligrams for the 10+ charge state (A,C) and the 9+ charge state (B,D) for WT (A,B) and A30P (C,D). Shaded regions represent the standard deviation (*n* = 3), and dashed lines indicate fits corresponding to distinct conformational populations.

### Osmolytes Alter the Conformational Dynamics of α-syn

Figure 3 illustrates the effects of TMAO and urea on the conformational dynamics of WT and A30P α-syn for the 9+ charge state with vT-ESI-IM-MS. Relative to WT without osmolyte (Figure 2B), both TMAO (Figure 3A) or urea (Figure 3B) shift the conformational distribution of WT toward more extended conformations. In addition, both osmolytes attenuate the temperature-dependent redistribution of conformers observed for WT alone. TMAO temperature-dependent population shifts are retained, showing fewer compact and intermediate conformers at 85 °C than at lower temperatures. In contrast, urea induces conformational population that remain nearly independent of temperature. In contrast, A30P in the presence of TMAO (Figure 3C) or urea (Figure 3D) exhibits a higher abundance of compact conformers than the corresponding WT samples. With TMAO, A30P shows increased compact conformers and reduced temperature-dependence. With urea, A30P displays a lower abundance of extended conformers relative to A30P alone and resembles the TMAO condition, except at 85 °C where urea yields a comparatively more extended conformation. At 85 °C, A30P with urea exhibits conformational populations similar to WT with urea; however, at lower temperatures, both variants show minimal temperature dependence, with A30P remaining more compact than WT. These differences between variants align with the different dynamics reported in previous studies.^48,51,52^ A similar trend is observed with the 10+ charge state (Figure S6) for TMAO and urea; mass spectra showed no observable difference (Figure S7) as well. Additionally, the mobiligrams for all charge states show no observable difference across temperatures (Figures S8 and S9).

**Figure 3:**
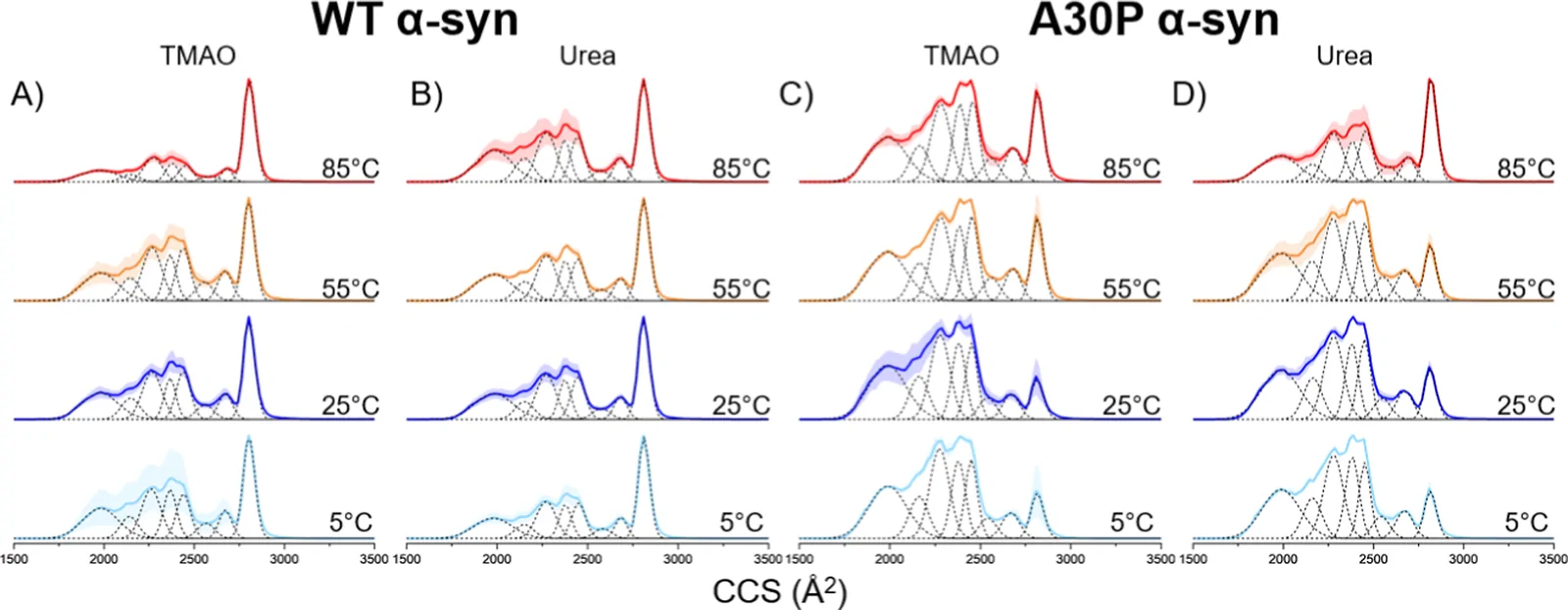
vT-ESI-IM-MS mobiligrams of the 9+ charge state of WT and A30P α-syn in 20 mM AmAc with 100 μM TMAO or urea at different ESI temperatures. Shown is the effect of TMAO (A,C) and urea (B,D) on WT (A,B) and A30P (C,D) α-syn. Mobiligrams were acquired at 5 (light blue), 25 (blue), 55 (orange), and 85 °C (red). Shaded regions represent the standard deviation (*n* = 3), and dashed lines indicate fits corresponding to distinct conformational populations.

To further probe osmolyte effects on α-syn conformational dynamics by vT-ESI-IM-MS, dodine, guanidine, and arginine, all containing guanidinium headgroups, were examined (Figure 4). For both WT and A30P in the presence of dodine (Figure 4A,D), minimal temperature dependence is observed; however, dodine promotes more extended WT conformations than are observed with no osmolyte (Figure 2B), or in the presence of TMAO (Figure 3A), and urea (Figure 3B). Similarly, A30P with dodine is more extended at lower temperatures compared to A30P without osmolyte (Figure 2D) and with TMAO (Figure 3C) or urea (Figure 3D) present. In contrast, both variants with guanidine (Figure 4B,E) or arginine (Figure 4C,F) exhibit nearly identical behavior, characterized by predominantly compact conformers and minimal temperature-dependent changes. Mass spectra across all temperatures, mobiligrams for the 10+ charge state, and all other charge states are provided in the Supporting Information for all the osmolytes tested (Figures S10–S14). Notably, no osmolyte-bound α-syn species were observed in the mass spectra with the presence of osmolytes, suggesting that the observed conformational changes arise from noncovalent interactions on the solvent environment and protein–solvent interactions rather than from binding. Osmolyte concentration produced distinct concentration-dependent changes in conformer populations (Figures S15): TMAO and dodine altered the abundance of compact conformers, whereas guanidine and arginine increased the population of extended conformers. Urea produced comparatively little concentration-dependent change. However, increasing osmolyte concentration substantially reduced ESI emitter stability and signal intensity, which limited the concentration range that could be reliably examined (Figure S15F).

**Figure 4:**
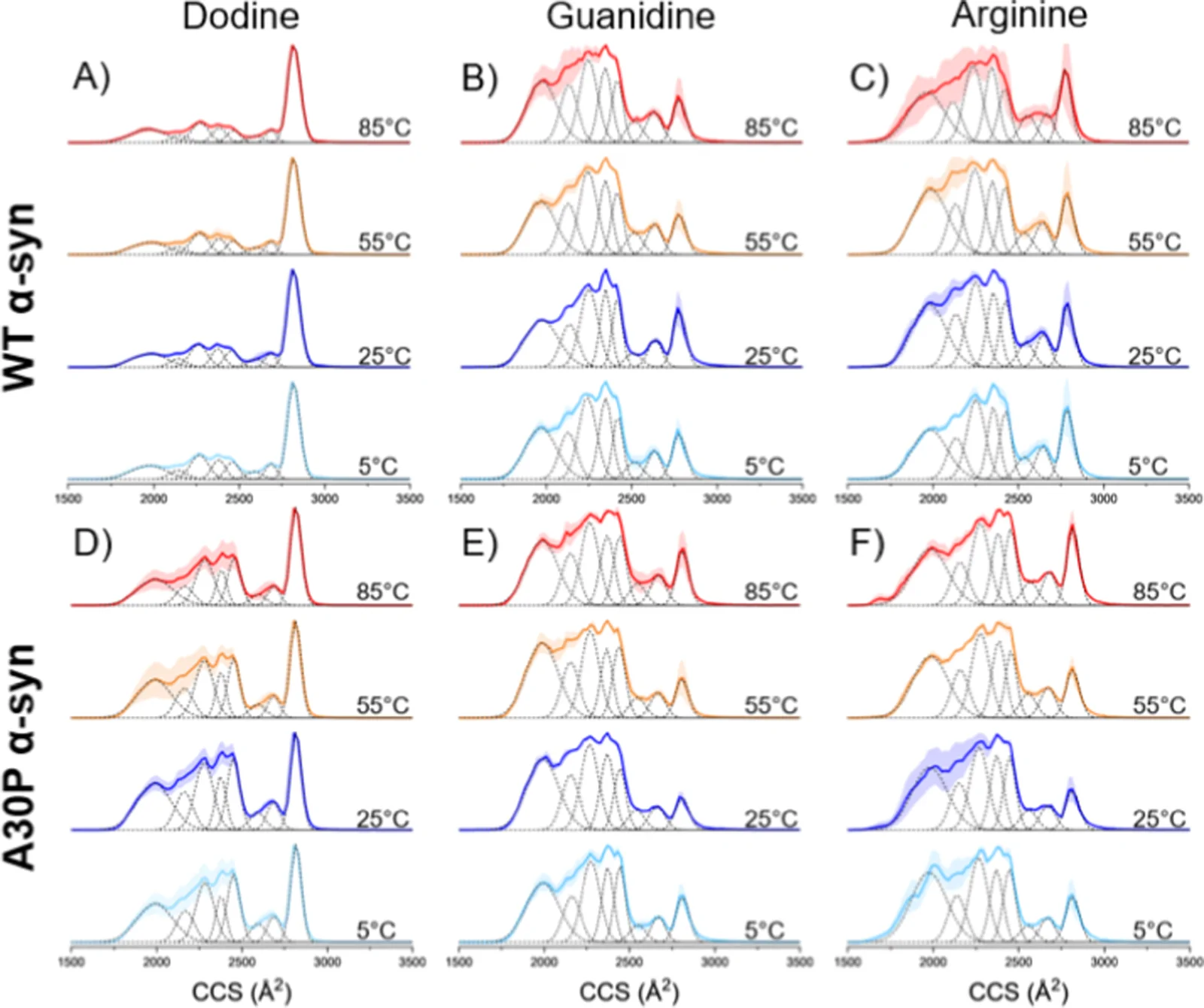
vT-ESI-IM-MS of the 9+ charge state of WT and A30P α-syn in 20 mM AmAc containing 100 μM of dodine, guanidine, or arginine at different ESI temperatures. Shown are WT (A–C) and A30P (D–F) α-syn with dodine (A,D), guanidine (B,E), and arginine (C,F). Mobiligrams were acquired at temperatures of 5 (light blue), 25 (blue), 55 (orange), and 85 °C (red). Shaded regions represent the standard deviation (*n* = 3), and dashed lines indicate fits corresponding to distinct conformational populations.

## Discussion

Previous mass spectrometry studies have investigated α-syn using IM-MS, hydrogen–deuterium exchange, chemical cross-linking, and electron capture dissociation.^53–57^ Building on these approaches, this work uses vT-ESI-IM-MS to study the misfolding behavior of WT and A30P α-syn demonstrating the utility of coupling IM-MS with vT-ESI to probe the conformational dynamics of IDPs. For globular proteins, thermal unfolding is often accompanied by distinct shifts in average charge state (*Z*_avg_), and similar changes have been reported upon ligand binding.^34^ These shifts are commonly interpreted as changes in solvent-accessible surface area (SASA) resulting from alterations in protein structure at higher osmolyte concentrations; however, such changes in *Z*_avg_ were not observed at the 100 μM osmolyte concentrations used in this study.^36,37,58^ In contrast, no significant differences in *Z*_avg_ were detected for α-syn as a function of temperature or osmolyte addition (Figure S16), indicating that these perturbations do not substantially alter the SASA of the protein. Despite the absence of measurable changes in *Z*_avg_, vT-ESI-IM-MS revealed pronounced temperature- and osmolyte-dependent shifts in conformational dynamics. These findings highlight the importance of ion mobility measurements, as the conformational dynamics of α-syn cannot be resolved from charge-state distributions alone.

Both WT and A30P α-syn exhibit similar temperature-dependent conformational dynamics for the 10+ and 9+ charge states (Figure 2), with compact conformers dominating at lower temperatures and extended conformers becoming increasingly abundant as temperature is raised, consistent with temperature-dependent population shifts.^50^ A similar finding can be observed in Figure S4 with the intensity of the 8+ charge state for WT α-syn changing drastically and the abundance of dimers decreasing for A30P which also indicate a change in conformational state. These shifts are likely driven by changes in protein–water interactions that modulate α-syn unfolding and conformational dynamics.^40^ Additionally, this can be interpreted to mean that the intramolecular contacts of the N-terminal region are lost at elevated temperatures. This matches previous studies and is observed with less extended conformers for A30P versus WT α-syn.^59^ Similar temperature-dependent behavior has been reported for other IDPs, including tau and casein, where altered solvent-mediated side-chain interactions distinguish IDPs from globular proteins.^60–62^ In the absence of osmolytes, increasing temperature shifts both WT and A30P α-syn toward more extended conformations, with A30P exhibiting a greater abundance of extended states than WT (Figures 2 and 5). Because extended conformers detected by IM-MS are generally associated with more non-native structures, this finding is noteworthy given that A30P displays a longer aggregation lag phase than WT despite forming aggregation-prone intermediates implicated in Parkinson’s disease.^51,52^ The increased population of extended conformers in A30P therefore likely reflects altered conformational dynamics rather than a direct increase in aggregation propensity. Similar temperature-dependent trends are also observed for the 10+ charge state (Figure S17).

**Figure 5:**
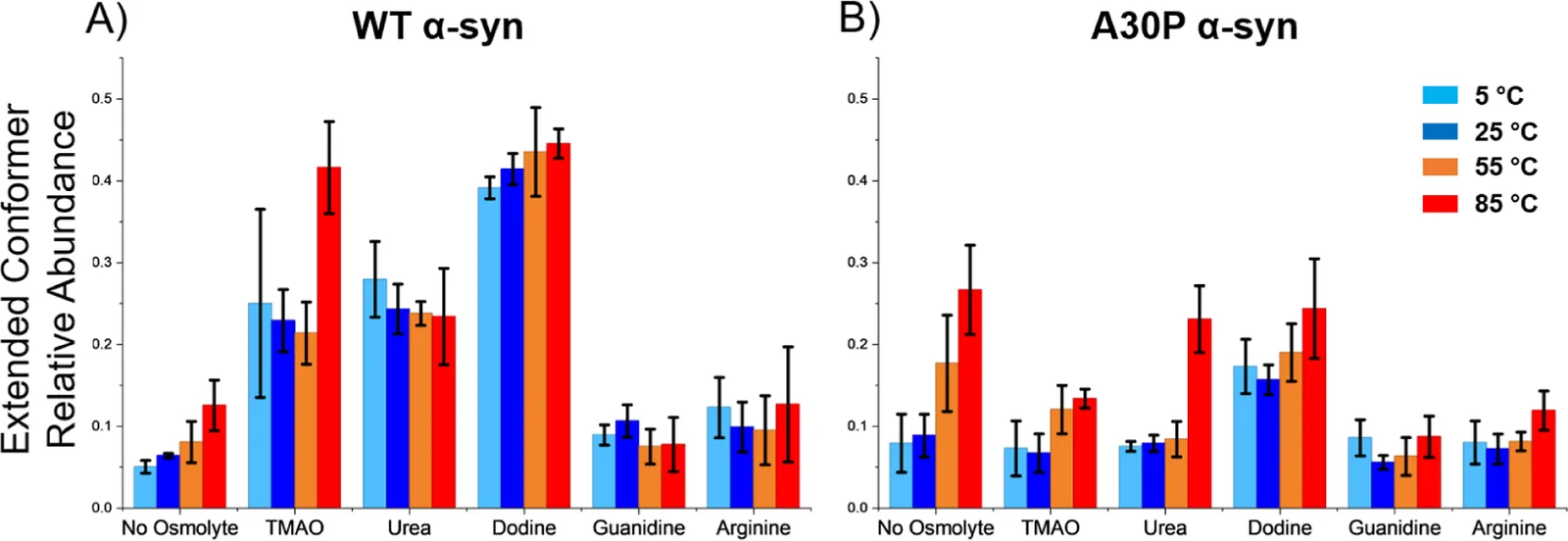
Relative abundance of the extended conformer for the 9+ charge state of WT and A30P α-syn measured by vT-ESI-IM-MS in the absence and presence of 100 μM osmolyte at different ESI temperatures. WT (A) and A30P (B) are shown without osmolyte and with TMAO, urea, dodine, guanidine, and arginine at temperatures of 5 (light blue), 25 (blue), 55 (orange), and 85 °C (red). Fitting parameters can be found in Figure S2 and Table S1 in the Supporting Information.

Osmolytes alter the conformational dynamics of WT α-syn, as reflected by changes in the relative abundance of the extended conformer across the range of ESI temperatures (Figure 5). Among the osmolytes examined, dodine produced the highest abundance of extended conformer and exhibits minimal temperature dependence, consistent with unfolding of α-syn. Because dodine preferentially interacts with an α-helical structure, these results may suggest the presence of an α helix on α-syn, as observed in membrane-bound α-syn.^13,63^ TMAO induced less conformational expansion than dodine but promoted increasingly extended conformations with increasing temperature. Urea exhibited a similar trend, although with a reduced temperature dependence and lower abundance of extended conformers relative to dodine. Interestingly, TMAO has also been reported to destabilize α-casein, another IDP, suggesting that osmolyte effects on IDPs may differ from those observed for globular proteins.^16^ This contrasts previous spectroscopic studies that show compaction with addition of TMAO; however this could be unique to the 100 μM of TMAO used as Figure S15A shows that compaction increases with higher concentration of TMAO.^19^ Interestingly, this effect is only observed for WT α-syn and not with A30P variant. The limited temperature dependence observed with dodine and urea suggests that both osmolytes reduce the conformational dynamics of WT α-syn relative to the no-osmolyte condition. In contrast, guanidine and arginine produce similar conformational distributions characterized by low abundances of extended conformers and minimal temperature dependence (Figure 4B,C). The response with arginine is consistent with its reported ability to suppress changes in protein dynamics, whereas the limited denaturing effect observed for guanidine may result from the relatively low concentration used in this study.^64,65^ The reduced temperature dependence on conformational dynamics is consistent with the excluded volume effect caused by osmolytes.^66^ That the osmolyte’s effects on α-syn did not align with previous literature can likely be attributed to the use of a micromolar rather than a molar concentration range of osmolyte.^17,18^ Overall, all osmolytes in this study attenuated the temperature-dependent conformational dynamics of WT α-syn as indicated by changes in the CCS conformational distribution. This is consistent with previous studies showing that osmolytes can alter protein folding temperatures and thermodynamic properties, an effect that has also been observed with the addition of methanol to the solution.^67–69^

The addition of osmolytes produced distinct conformational responses for A30P compared with WT α-syn (Figure 5). In the absence of osmolytes, A30P exhibits a higher abundance of the extended conformer than WT while maintaining a similar temperature-dependent shift. In contrast, TMAO, urea, and dodine reduce the abundance of the extended conformer more for A30P than for WT. Notably, TMAO also decreases the abundance of the extended conformer relative to osmolyte-free A30P, a trend not observed for WT. Guanidine and arginine yield similar conformational distributions for both variants, suggesting that their effects are less sensitive to the A30P substitution. These differences may arise from the substitution of proline residues can undergo a cis–trans isomerization that alter the protein dynamics.^70–72^ The reduced abundance of the extended conformation observed for A30P in the presence of TMAO, urea, and dodine suggests that the conformational states stabilized by these osmolytes in WT α-syn are altered by the A30P substitution. A possible explanation is that these osmolytes are perturbing the N-terminal region of α-syn, whereas with the A30P variant the dynamics of this region are modified via altered hydration, reducing its susceptibility to osmolyte-induced changes. These differences are further highlighted in Figure S18, where the relative abundance of the extended conformer is significantly altered by TMAO, urea, and dodine across all temperatures for WT α-syn but not for A30P. Moreover, WT consistently exhibits a greater abundance of the extended conformer than A30P in the presence of osmolytes, suggesting that the A30P substitution attenuates osmolyte-induced changes in conformational dynamics. Importantly, no osmolyte was observed to bind to WT or A30P α-syn, indicating that the observed changes arise from noncovalent modulation of protein–solvent interactions rather than direct osmolyte binding.^9^

The effects of osmolytes on the conformational distributions of α-syn can be interpreted in terms of their influence on the surrounding water network.^73^ Osmolytes are often classified as kosmotropes or chaotropes based on their ability to promote or disrupt water structure, respectively.^74^ Within this framework, the higher abundance of the extended conformer observed in the presence of TMAO, urea, and dodine, compared to guanidine and arginine, suggests differences in how these osmolytes modulate protein–solvent interactions. However, as discussed by Lee and Sapienza, these effects may be more appropriately interpreted in terms of protein dynamics rather than water structure-making or structure-breaking.^40^ Notably, the same number of conformers was observed across the CCS distributions, indicating that the conformational heterogeneity of the ensemble remained consistent, while the relative abundances of the individual conformers changed with temperature and osmolyte conditions. These changes in conformer populations reflect redistribution among existing conformational states and are therefore interpreted as changes in conformational dynamics. Examination of the CCS distributions in Figures 2–4 indicates that guanidine and arginine maintain a larger population of compact conformers while exhibiting heterogeneous CCS distributions; this suggests that these osmolytes preserve a broader ensemble of compact and intermediate conformational states. In contrast, TMAO, urea, and dodine shift the conformational ensemble toward more extended states, resulting in a reduced relative population of compact and intermediate conformers. These results suggest that osmolyte-dependent changes in α-syn physicochemical properties is better described by the effect on protein dynamics and hydration rather than by the traditional kosmotrope-chaotrope classification alone. It is important to note that the results observed here differ from that reported in many previous osmolyte studies, which commonly employ osmolyte concentrations ranging from millimolar to molar levels.^12,17,19,65^ The comparatively low osmolyte concentrations used in this study may therefore contribute to the distinct conformational behavior observed by vT-ESI-IM-MS.

## Conclusion

Variable temperature ESI-IM-MS was used to investigate the effects of osmolytes on the conformational dynamics of WT and A30P α-syn. The differences induced by osmolyte addition can be interpreted in the context of the dynamic model proposed by Lee et al., in which perturbations alter the dynamics of specific regions within an IDP.^40^ The increase in the abundance of extended conformers for WT in the presence of TMAO, urea, and dodine compared to A30P indicates a decrease in the conformational dynamics of WT α-syn relative to A30P. Comparing these extended conformer abundances further suggests that the A30P mutation perturbs osmolyte-induced changes in dynamics. This indicates that TMAO, urea, and dodine preferentially alter the N-terminal region, resulting in an increase in both extended conformers and dynamics. In contrast, guanidine and arginine favor more compact conformers with reduced temperature dependence exhibiting similar responses for both WT and A30P α-syn. The minimal differences observed between WT and A30P in the presence of guanidine and arginine with the changes introduced by the A30P mutation, or that they modulate α-syn through a different domain.

These results demonstrate the utility of osmolytes as probes for IDP conformational dynamics as shown by changes in the abundance of conformer distributions through noncovalent modulation of protein–solvent interactions.^9^ Although this work focused on the effects of osmolytes on the conformational dynamics of α-syn, this experimental strategy could be extended to investigate the influence of ligand or metal binding on α-syn dynamics.^28,75^ Further studies could also examine additional disease-associated α-syn variants to determine how familial mutations alter osmolyte-dependent conformations.^76^ Furthermore, this methodology may be applied to other IDPs, including tau and amyloid-β, to investigate the effects of environmental perturbations on their dynamics.^25,77^ Osmolytes also serve as biomarkers of various disease states through their altered regulation, and the present work provides insight into how changes in osmolyte composition may modulate the conformational dynamics and stability of proteins across the proteome.^78,79^ More broadly, these findings underscore that the conformational dynamics of IDPs are strongly influenced by their chemical environment and suggest that altering solvent conditions can selectively stabilize conformational states involved in disease-associated aggregation, consistent with observations from limited proteolysis experiments.^80^

## Supplementary Material



## Notes

Images of the variable-temperature device, representative fitting procedures and parameters, additional vT-ESI-IM-MS mass spectra and mobiligrams, *Z*_avg_, and relative abundance conformer population analyses for the 10+ charge state (PDF)

## †

Department of Chemistry and Biochemistry, The University of Oklahoma, Norman, Oklahoma, 73019
