# Supplementary material for: Influence of Temperature and Osmolytes on Conformational Dynamics of Alpha Synuclein
Source: J Phys Chem B. 2026 Oct 13;130(42):11122–31. doi: 10.1021/acs.jpcb.6c04859 (PMC13700001; doi:10.1021/acs.jpcb.6c04859)
Supplement: Supplementary file 1 [file jp6c04859_si_001.pdf]

Supporting Information for:

Influence of Temperature and Osmolytes on Conformational Dynamics of Alpha Synuclein

Jared Hampton, Cameron Lynch, Robert L. Rider, Carter Lantz, Zhenyu Xi, David H. Russell*

Department of Chemistry, Texas A&M University, College Station, Texas 77843

*Corresponding Author, email: russell@chem.tamu.edu

Table of Contents Page

Table of Contents	Page
Figure S1	S2
Figure S2	S2
Figure S3	S3
Table S1	S3
Figure S4	S4
Figure S5	S4
Figure S6	S5
Figure S7	S5
Figure S8	S6
Figure S9	S6
Figure S10	S7
Figure S11	S7
Figure S12	S8
Figure S13	S8
Figure S14	S9
Figure S15	S9
Figure S16	S10
Figure S17	S10
Figure S18	S11
Python Script	S11

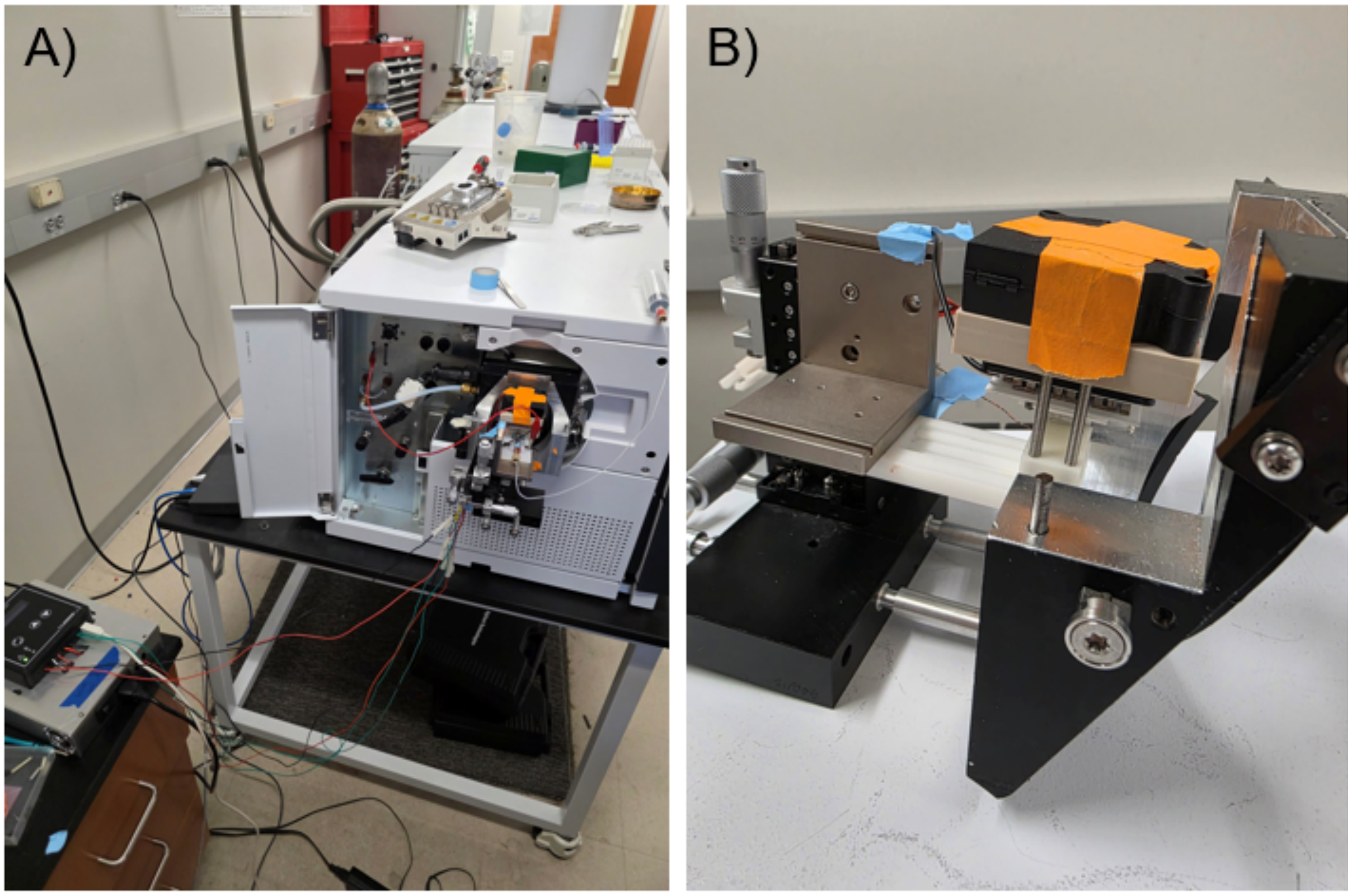

Figure S1: (A) The Agilent 6560 IM-Q-TOF ion mobility-mass spectrometer (IM-MS) system with a variable temperature device connected to the control box. (B) Side view of the variable-temperature device.

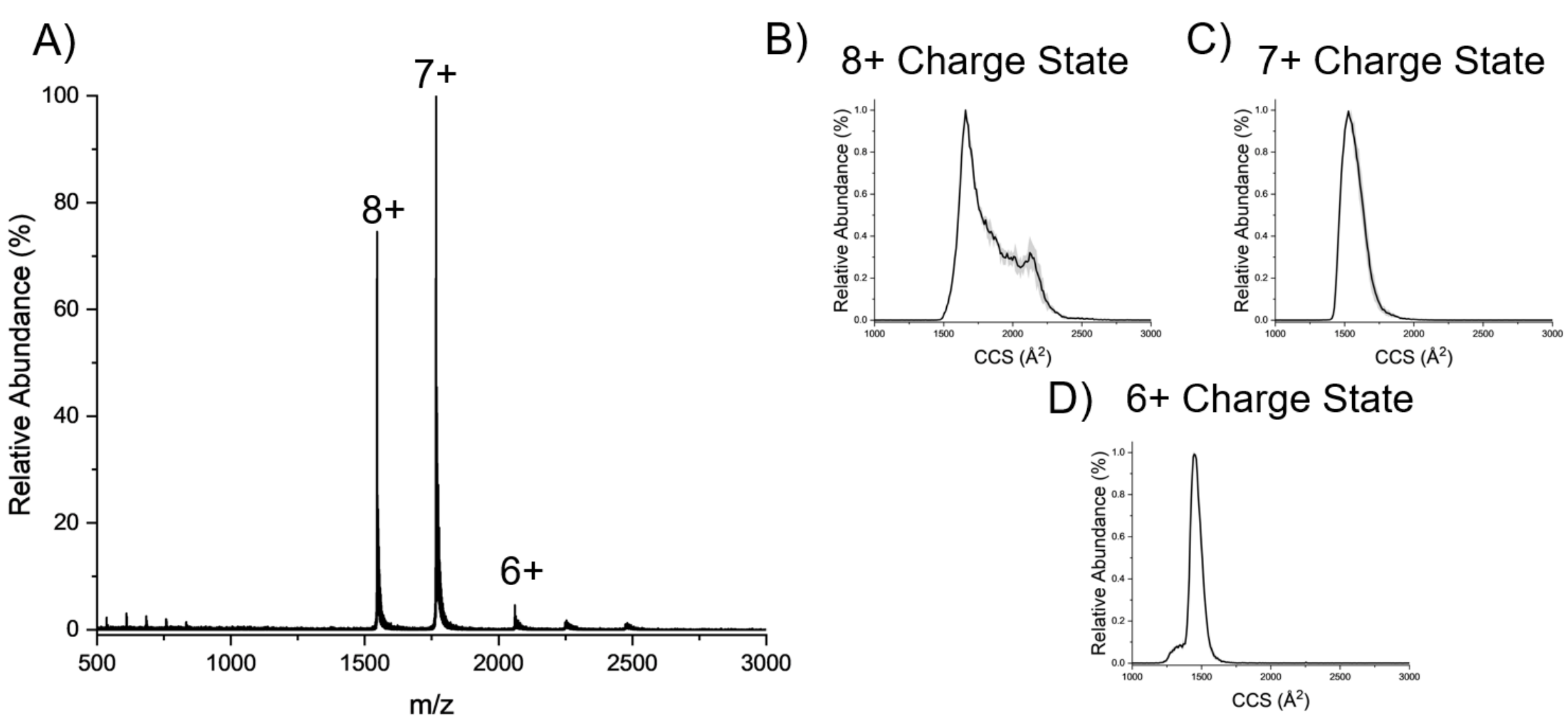

Figure S2: Native mass spectra and ion mobiligrams of 10 μM cytochrome c. Shown are the native mass spectrum (A) and corresponding mobiligrams for the 8+ (B), 7+ (C), and 6+ (D) charge states.

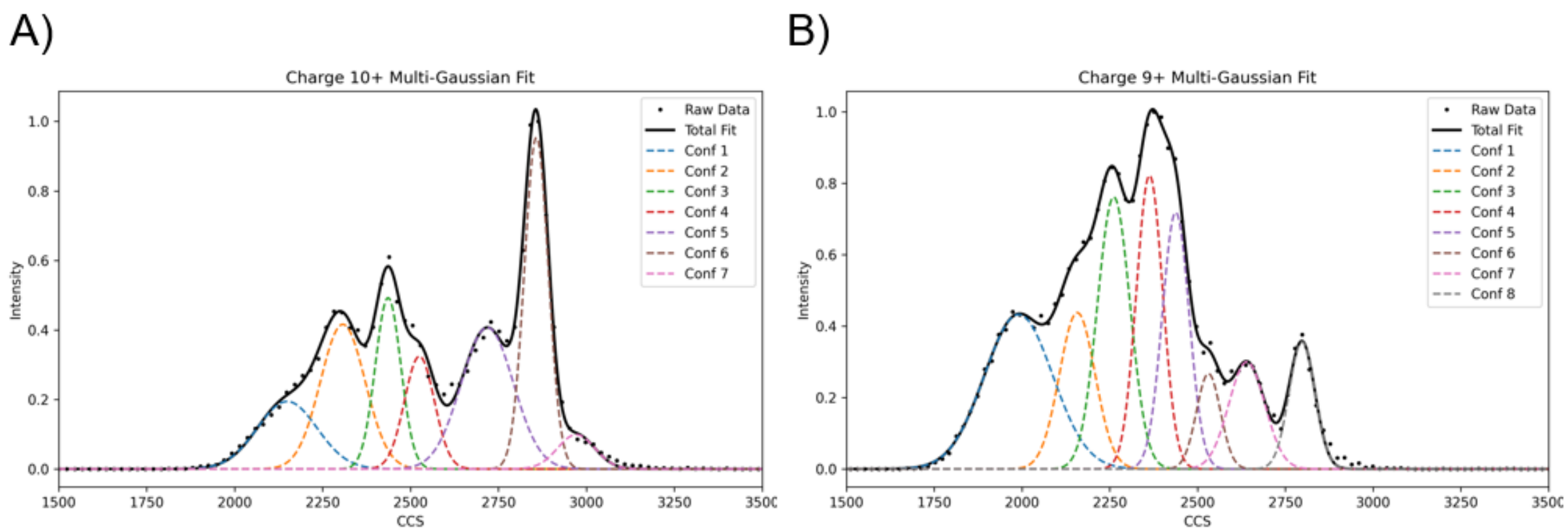

Figure S3: Deconvoluted charge states of WT α-syn for the 10+ and 9+ charge states. Shown is the 10+ charge state fit to 7 individual conformers (A) and the 9+ charge state fit to 8 individual conformers (B).

Table S1: Gaussian fitting information for the 10+ and 9+ charge states found in Figure S2.

Charge State	Conf #	CCS Bounds (Å^2)	FWHM Bounds (Å^2)
10+	1	1,700 – 2,150	2 – 200
	2	2,250 – 2,400	2 – 150
	3	2,400 – 2,500	2 – 125
	4	2,500 – 2,600	2 – 100
	5	2,700 – 2,800	2 – 175
	6	2,800 – 2,900	2 – 100
	7	2,950 – 3,100	2 – 125
9+	1	1,900 – 2,000	20 – 250
	2	2,100 – 2,200	2 – 120
	3	2,200 – 2,300	2 – 125
	4	2,300 – 2,400	2 – 100
	5	2,400 – 2,500	2 – 90
	6	2,500 – 2,600	2 – 110
	7	2,600 – 2,700	2 – 125
	8	2,750 – 2,900	2 – 100

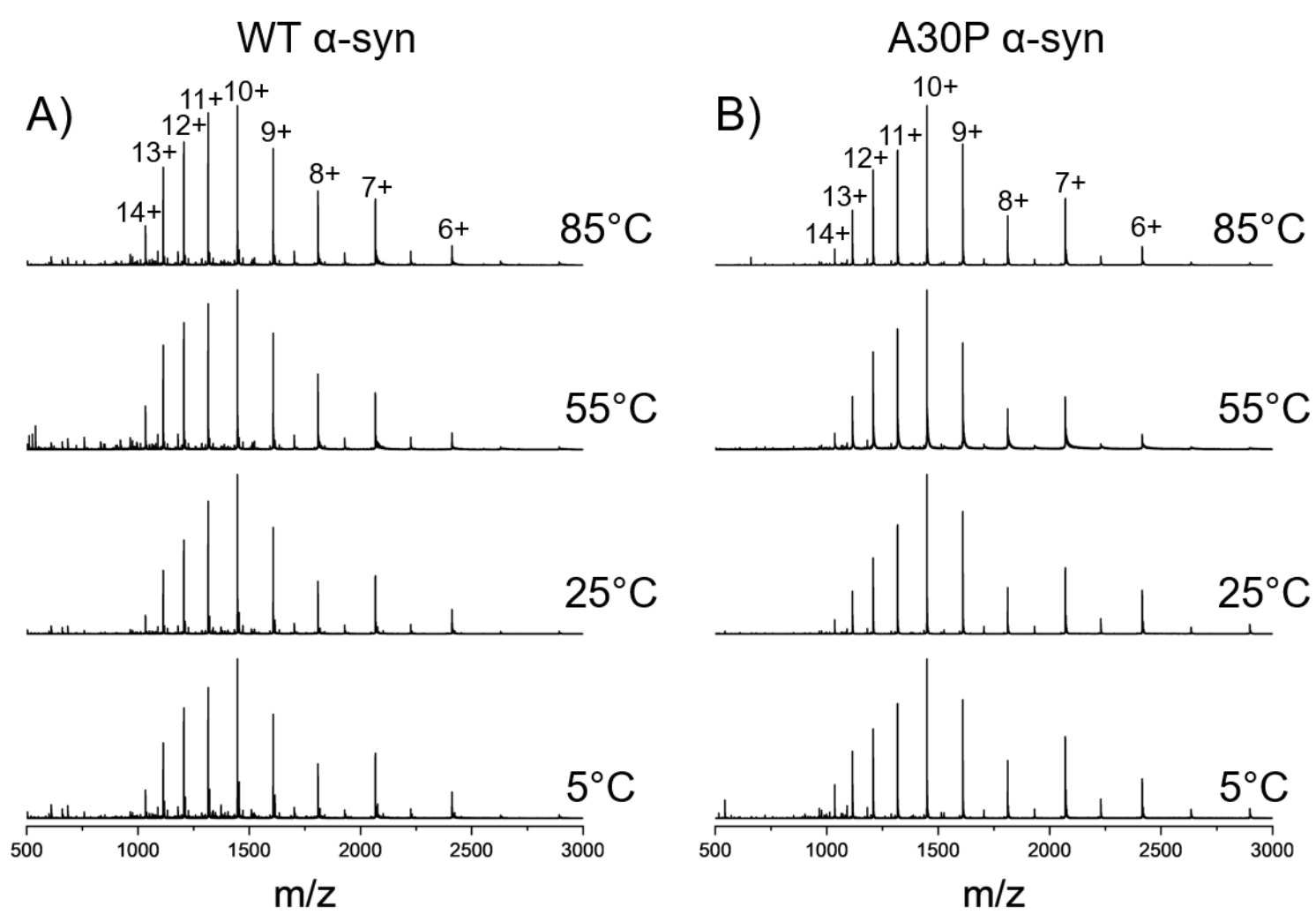

Figure S4: vT-ESI-IM-MS mass spectra of WT (A) and A30P (B) α-syn in the absence of osmolyte, at temperatures of 5, 25, 55, and 85 °C (bottom to top).

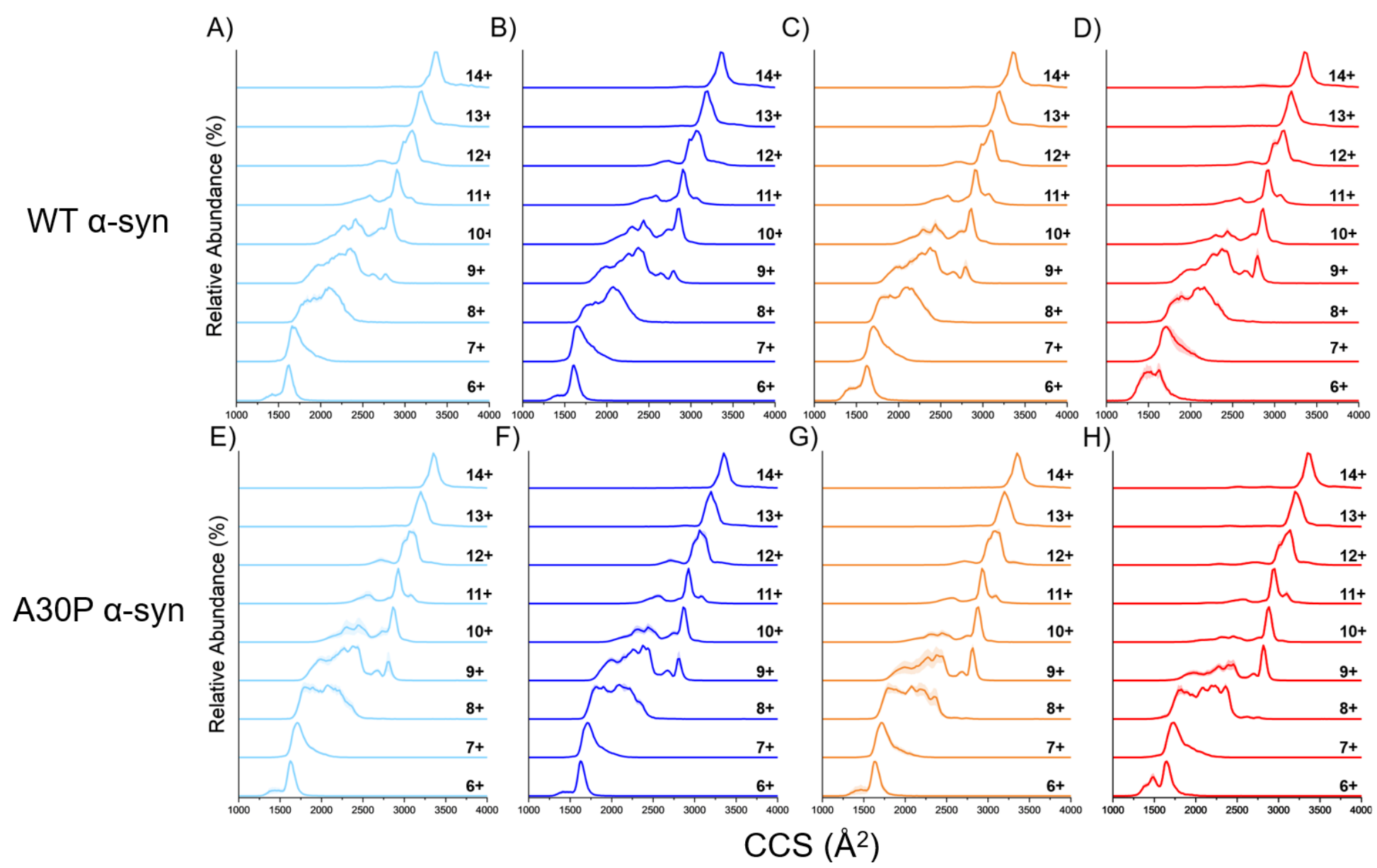

Figure S5: vT-ESI-IM-MS mobiligrams for charge states 14+ to 6+ for WT (A-D) and A30P (E-H) α-syn without osmolyteat temperatures of 5 (light blue; A, E), 25 (blue; B, E), 55 (orange; C, G), and 85 °C (red; D, H).

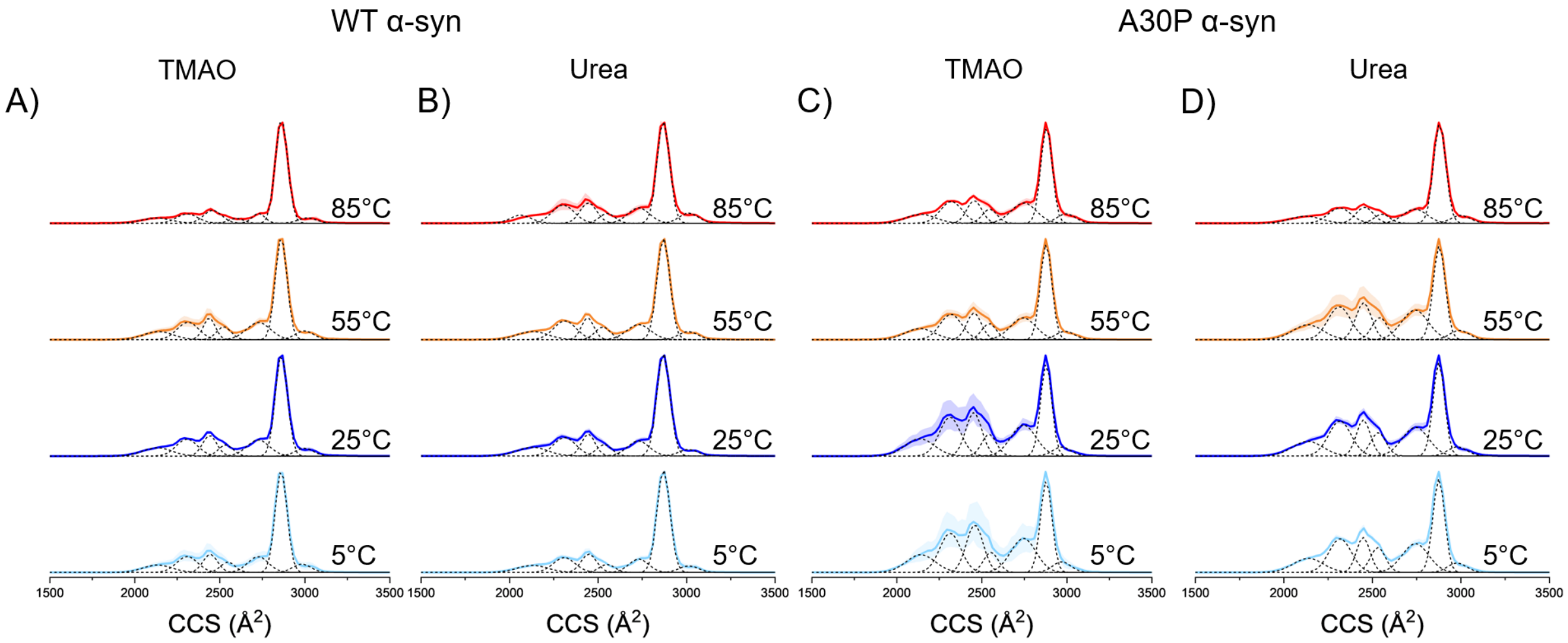

Figure S6: vT-ESI-IM-MS mobiligrams of the 10+ charge state of α-syn (WT and A30P) in 20 mM AmAc with 100 µM TMAO or urea at different ESI temperatures: (A) and (B) are for WT a-syn with TMAO and urea, respectively; (C) and (D) are for A30P a-syn with TMAO and urea, respectively. Mobiligrams were acquired at temperatures of 5 (light blue), 25 (blue), 55 (orange), and 85 °C (red). Shaded regions represent the standard deviation (n=3), and dashed lines indicate fits corresponding to distinct conformational populations.

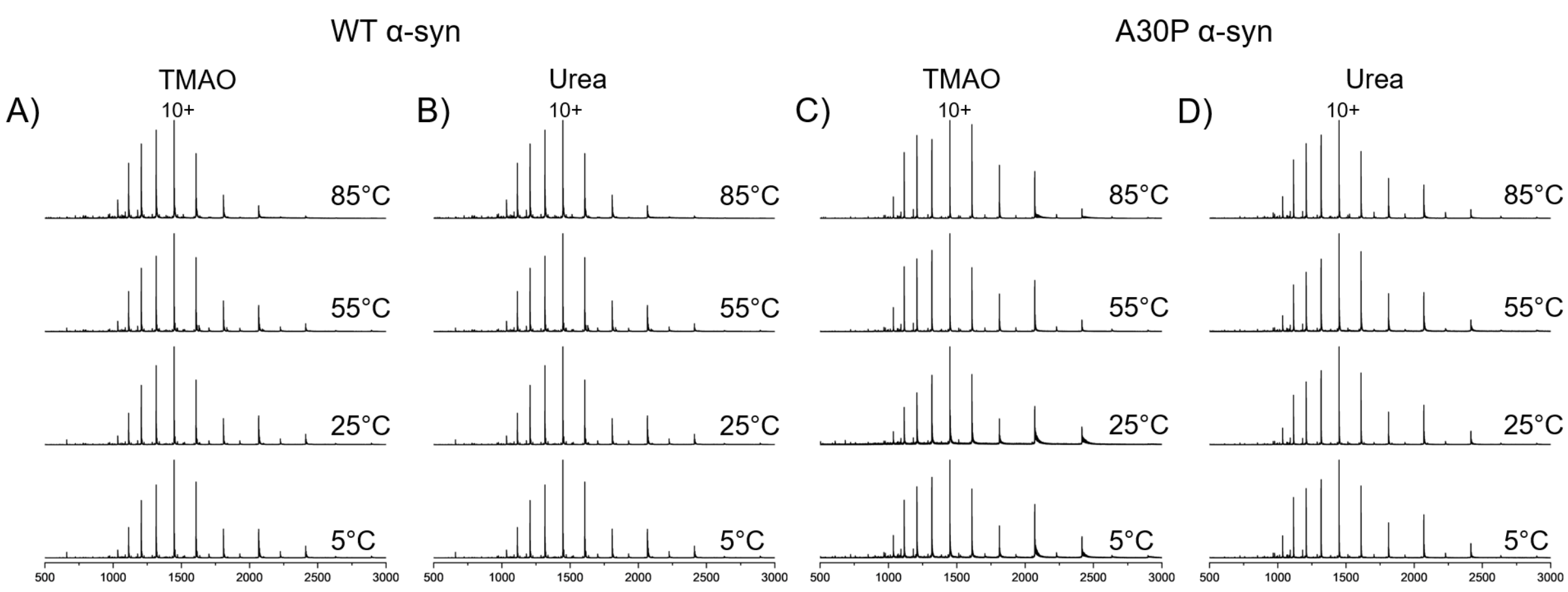

Figure S7: vT-ESI-IM-MS mass spectra at temperatures of 5, 25, 55, and 85 °C (bottom to top) of WT a-syn with 100 uM of (A) TMAO and (B) urea; (C) and (D) are spectra of A30P α-syn with 100 µM of TMAO and urea, respectively.

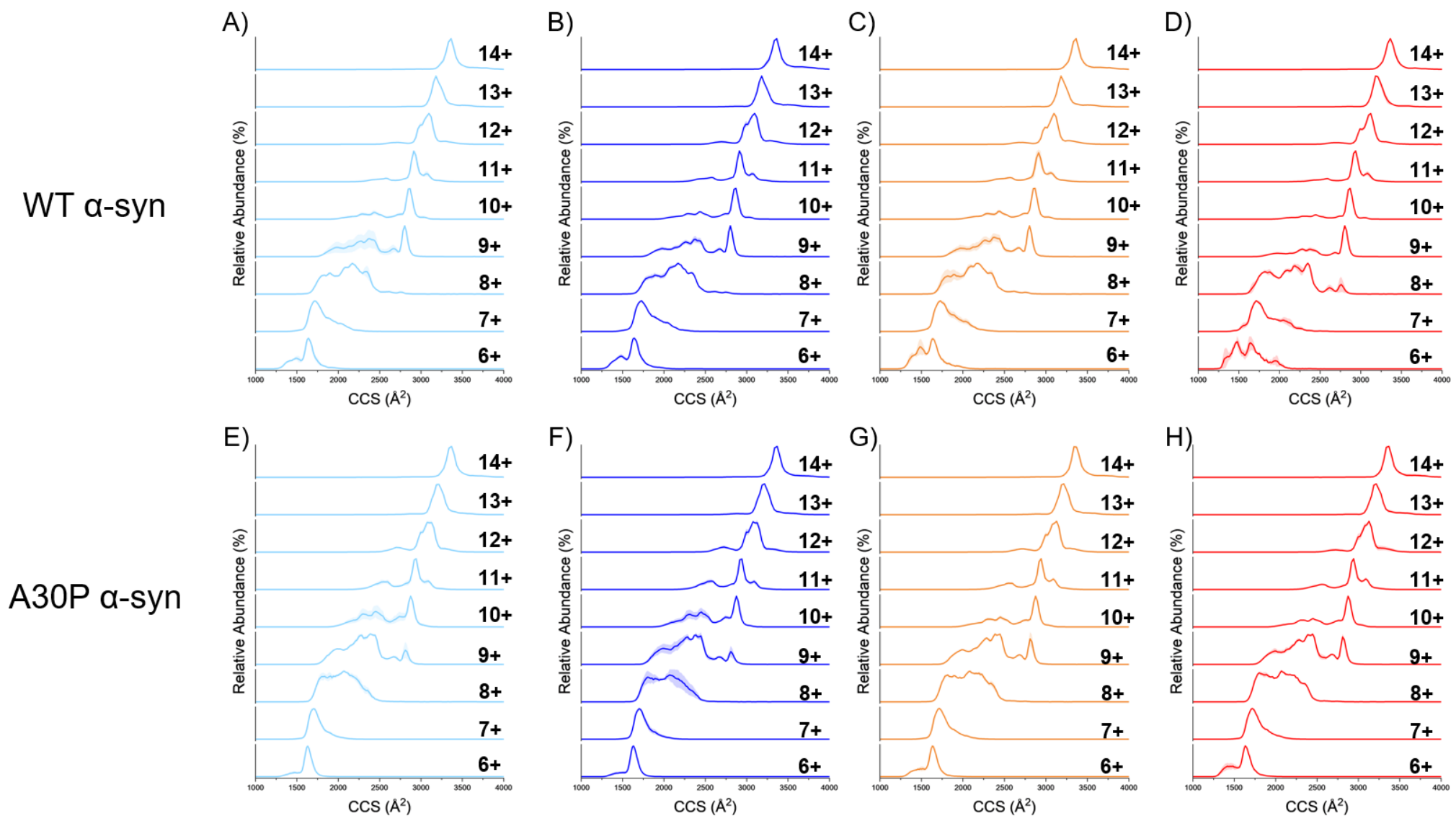

Figure S8: vT-ESI-IM-MS mobiligrams for charge states 14+ to 6+ of α-syn WT (A-D) and A30P (E-H) with 100 µM TMAO at temperatures of 5 (light blue), 25 (blue) 33 (orange) and 85 °C (red).

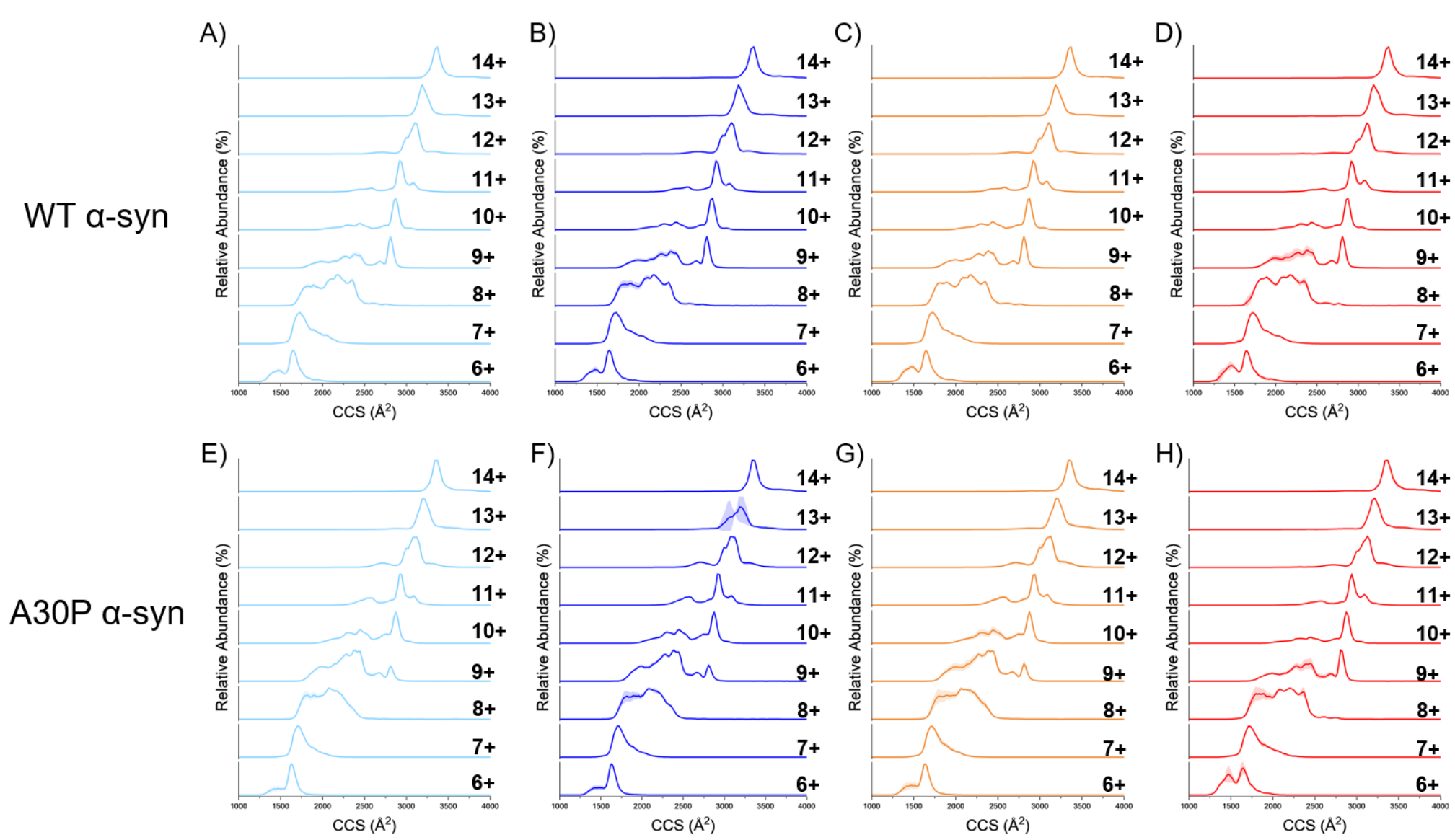

Figure S9: vT-ESI-IM-MS mobiligrams for charge states 14+ to 6+ of α-syn WT (A-D) and A30P (E-H) with 100 µM urea at temperatures of 5 (light blue), 25 (blue) 33 (orange) and 85 °C (red).

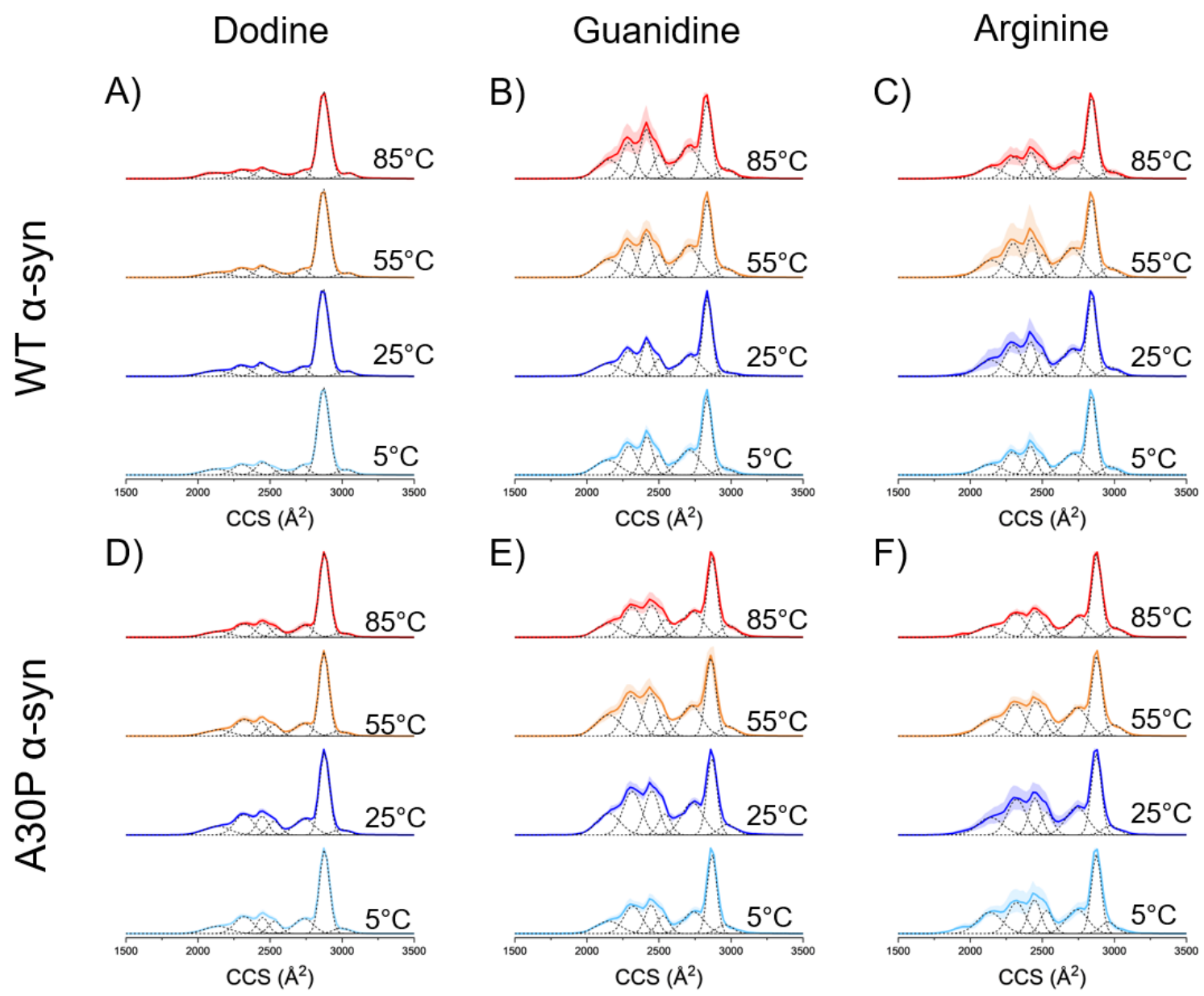

Figure S10: vT-ESI-IM-MS mobiligrams of the 10+ charge state of WT and A30P α-syn in 20 mM AmAc acquired at temperatures of 5 (light blue), 25 (blue), 55 (orange), and 85 °C (red): WT α-syn with 100 mM of (A) dodine; B (guanidine); C (arginine);A30P α-syn with 100 μM of (D) dodine; (E) guanidine; (F)Shaded regions represent the standard deviation (n=3), and dashed lines indicate fits corresponding to distinct conformational populations.

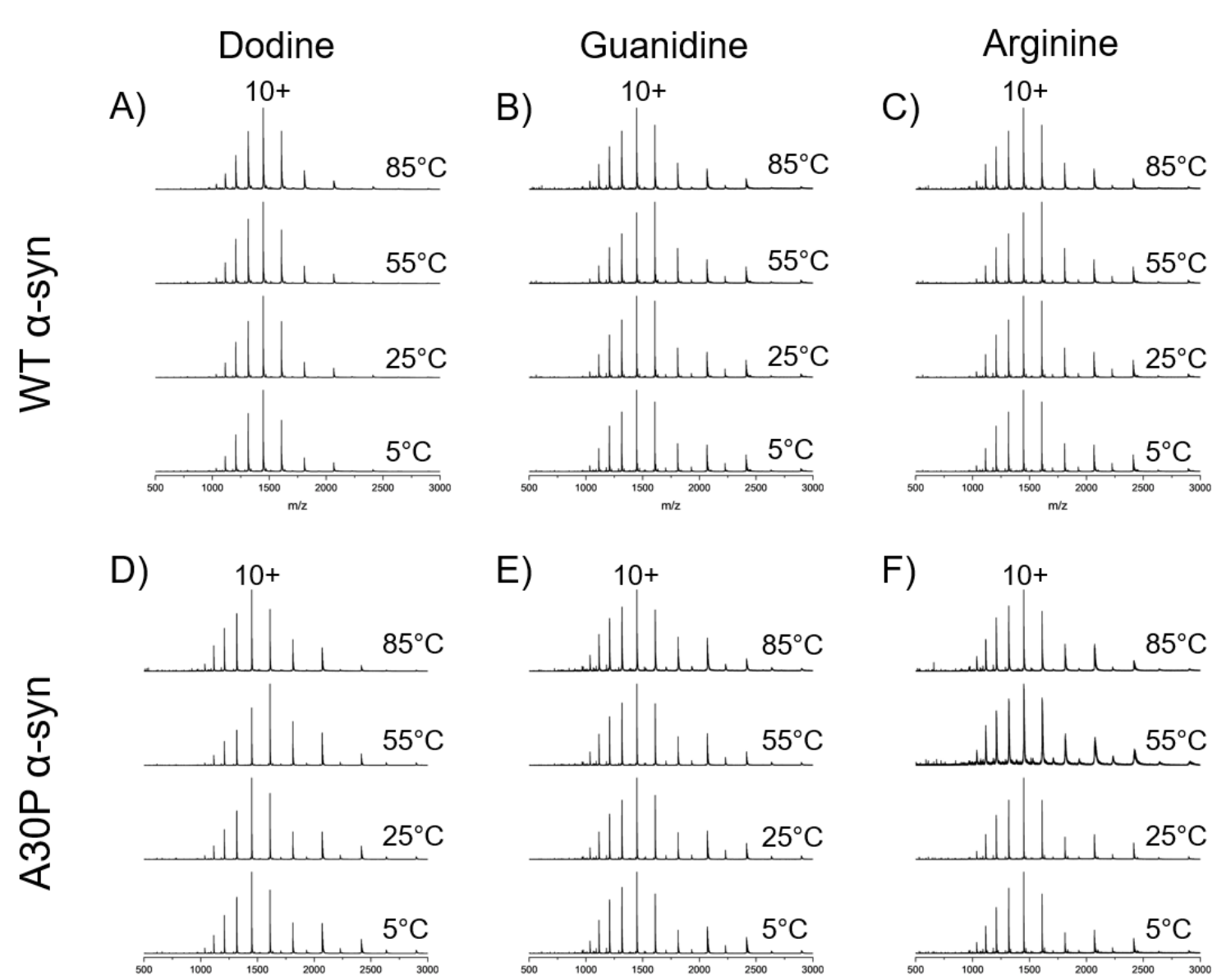

Figure S11: vT-ESI-IM-MS mass spectra of WT and A30P α-syn with 100 μM dodine, guanidine, or arginine: Shown is WT (A) and A30P (B) with dodine (A, D), guanidine (B, E), and arginine (C, F) at temperatures of 5, 25, 55, and 85 °C (bottom to top).

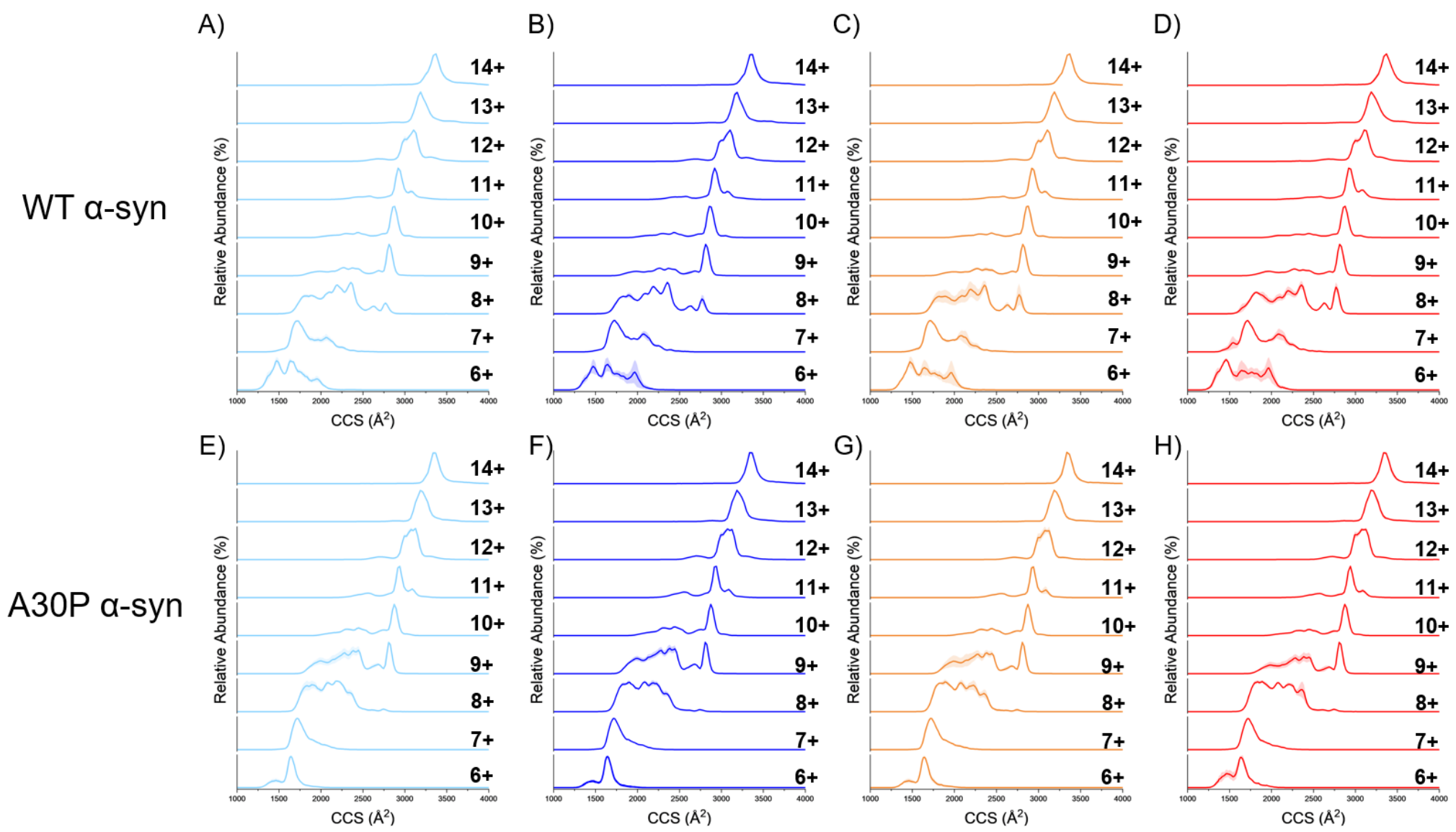

Figure S12: vT-ESI-IM-MS mobiligrams for charge states 14+ to 6+ of α-syn WT (A-D) and A30P (E-H) with 100 μM dodine at temperatures of 5 (light blue), 25 (blue) 33 (orange) and 85 °C (red).

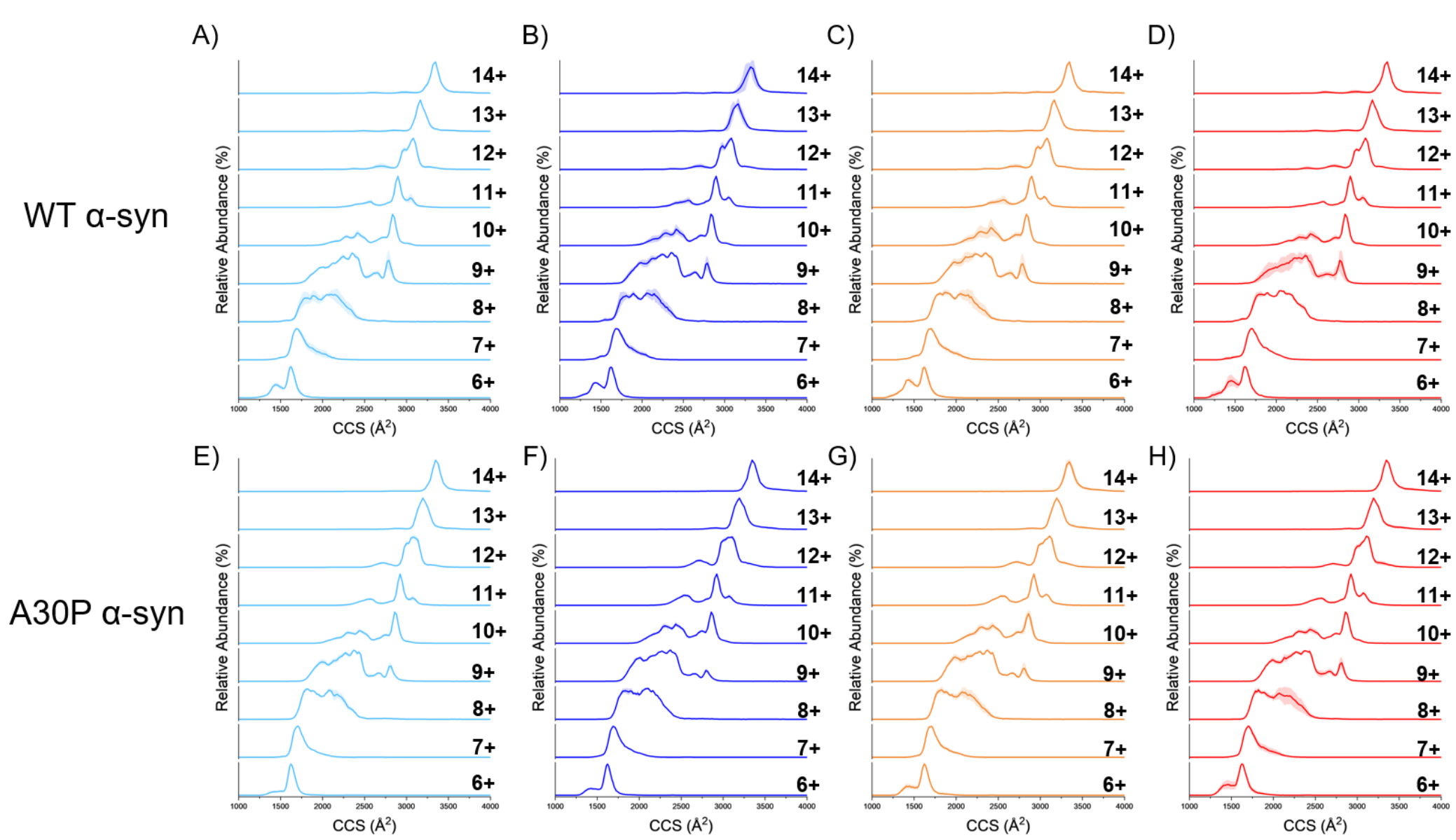

Figure S13: vT-ESI-IM-MS mobiligrams for charge states 14+ to 6+ of α-syn WT (A-D) and A30P (E-H) with 100 μM guanidine at temperatures of 5 (light blue), 25 (blue) 33 (orange) and 85 °C (red).

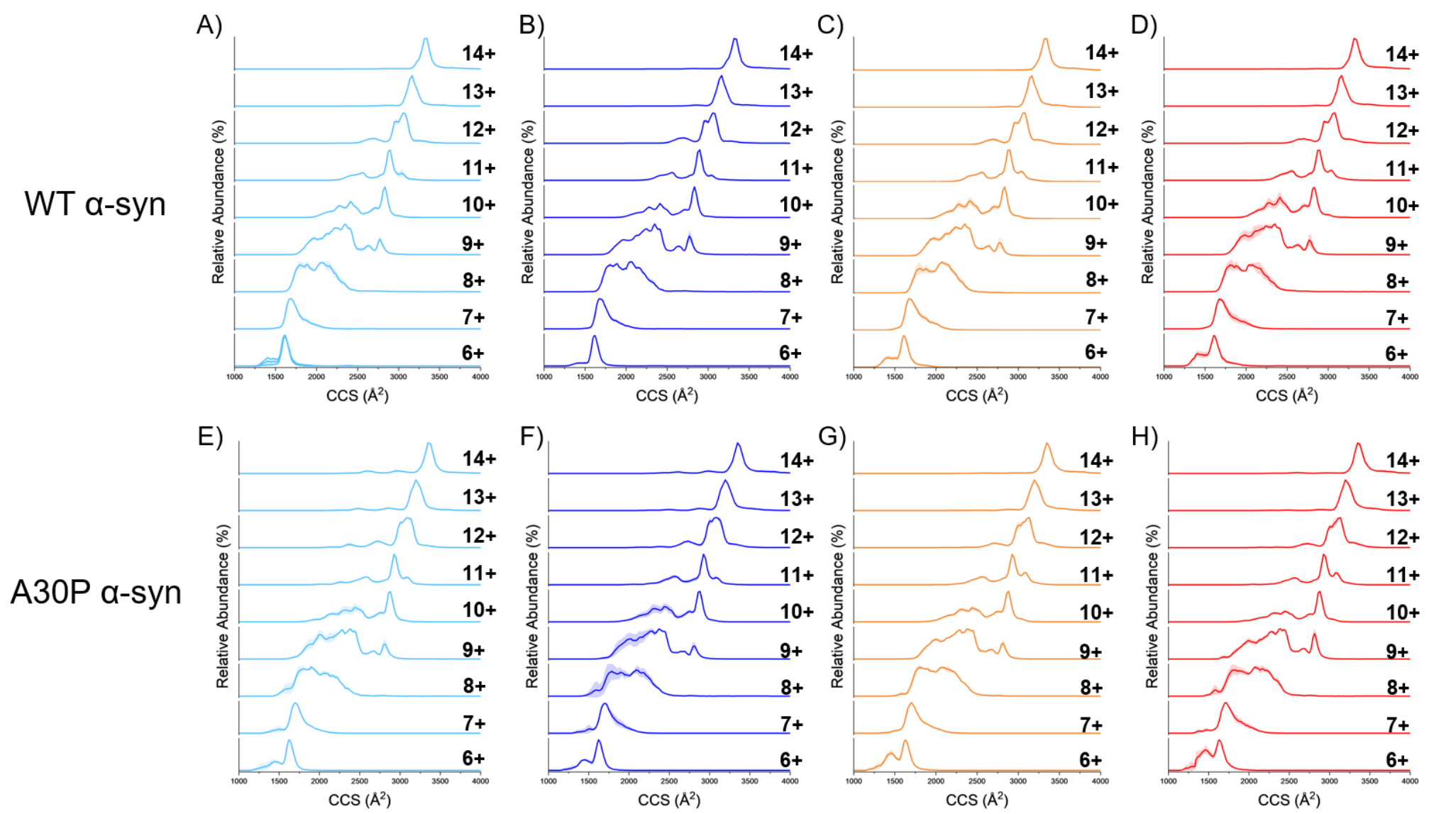

Figure S14: vT-ESI-IM-MS mobiligrams for charge states 14+ to 6+ of α-syn WT (A-D) and A30P (E-H) with 100 μM arginine at temperatures of 5 (light blue), 25 (blue) 33 (orange) and 85 °C (red).

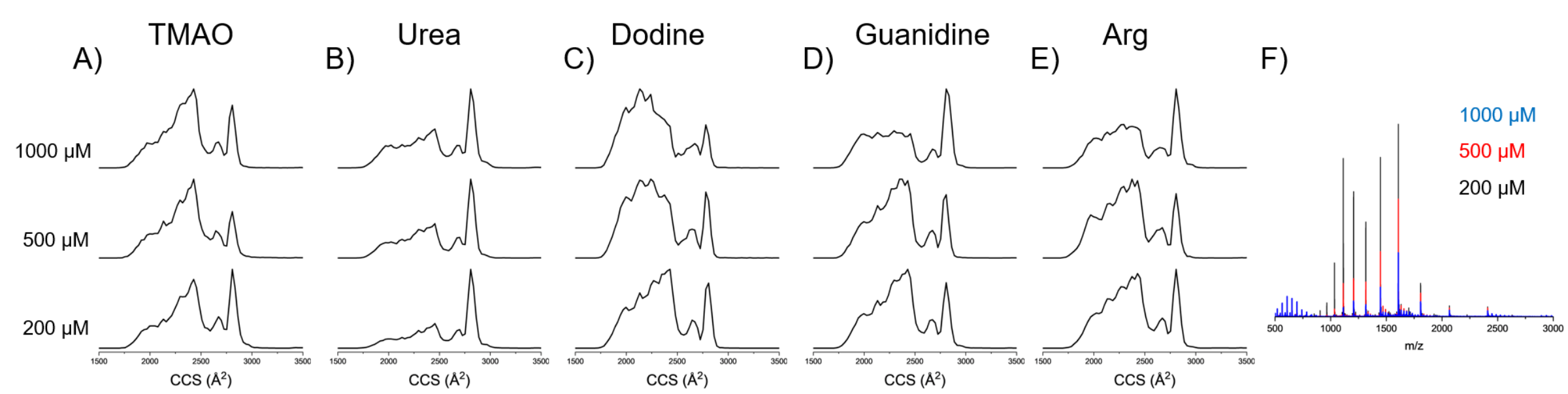

Figure S15: IM-MS mobiligrams of the 9+ charge state for WT α-syn with various concentrations of osmolytes and representative mass spectra. Mobiligrams are shown for 200 (bottom), 500 (middle), and 1000 (top) μM TMAO (A), urea (B), dodine (C), guanidine (D), and arginine (E). Representative mass spectra of WT α-syn with 200 (black), 500 (red), and 1000 (blue) μM dodine show decreasing signal intensity with increasing osmolyte concentration.

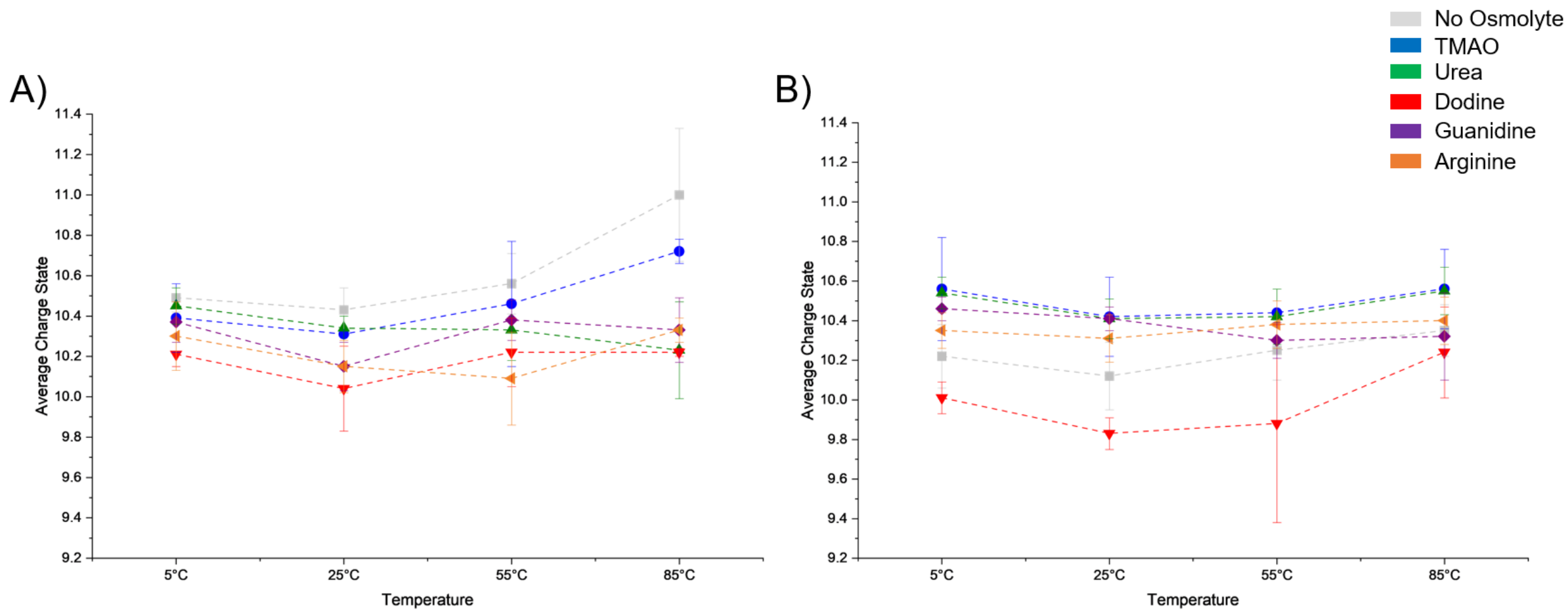

Figure S16: Average charge states of WT and A30P α-syn measured by vT-ESI-IM-MS with and without osmolytes at varying temperatures. WT (A) and A30P (B) average charge state graphs are shown without osmolyte (grey) and with 100 μM TMAO (blue), urea (green), dodine (red), guanidine (purple), and arginine (orange) at temperatures of 5, 25, 55, and 85 °C.

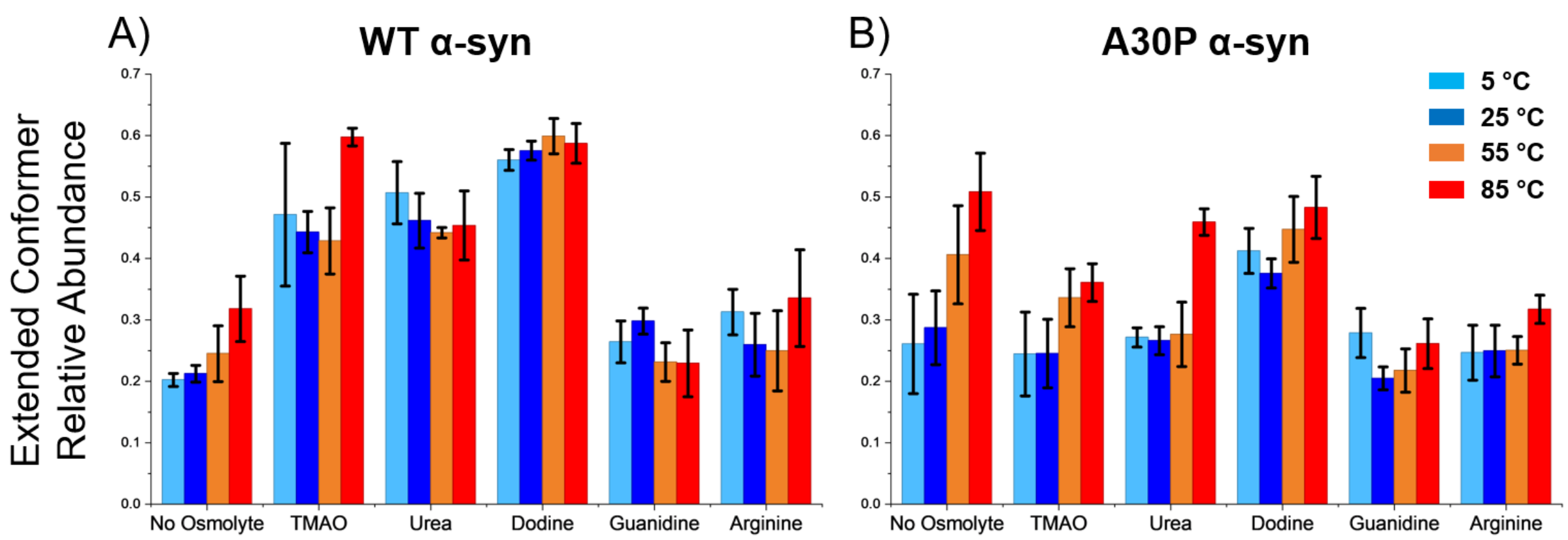

Figure S17: Mole fraction of the extended conformer for the 10+ charge state of WT and A30P α-syn measured by vT-ESI-IM-MS in the absence and presence of 100 μM osmolyte at different ESI temperatures. WT (A) and A30P (B) are shown without osmolyte and with TMAO, urea, dodine, guanidine, and arginine at temperatures of 5 (light blue), 25 (blue), 55 (orange), and 85 °C (red).

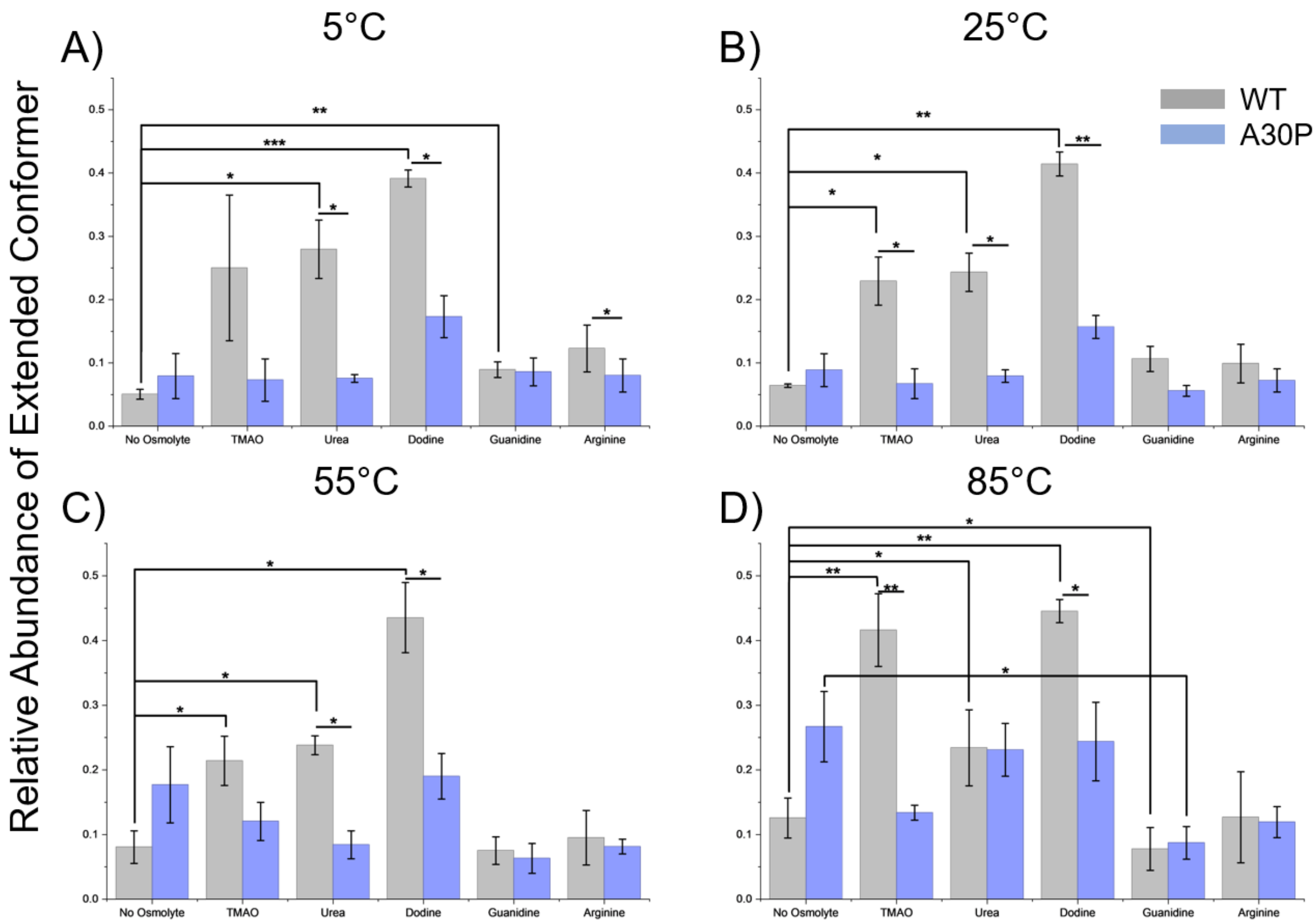

Figure S18: Extended conformer mole fraction of the 9+ charge state of WT and A30P α-syn measured by vT-ESI-IM-MS at different temperatures. WT (grey) and A30P (blue) are shown at temperatures of 5 (A), 25 (B), 55 (C), and 85 °C (D) in the absence of osmolyte and in the presence of 100 μM TMAO, urea, dodine, guanidine, and arginine. Error bars represent the standard deviation (n=3). Statistical significance is indicated as $p < 0.05$ (*), $p < 0.01$ (**), and $p < 0.001$ (***).

Python Script

```
"""
Multi-Gaussian fitting script for CCS vs intensity data.

- Input: two-column text file (no header): CCS intensity
- Fits multiple Gaussian peaks simultaneously (total summed fit)
- Outputs fitted parameters to 'output.txt'
- Displays total fit and individual components

Usage:
    python Conformer_Fit.py --charge 7 --input input.txt #Read this in the future
so that you can get this to work
```

```
"""

import argparse
import numpy as np
import matplotlib.pyplot as plt
from scipy.optimize import curve_fit

# -------------------------
# Gaussian and total function
# -------------------------
def gaussian(x, amp, mean, fwhm):
    sigma = fwhm / 2.354820045
    return amp * np.exp(-0.5 * ((x - mean) / sigma) ** 2)

def multi_gaussian(x, *params):
    """Sum of multiple Gaussians. Params = [amp1, mean1, fwhm1, amp2, mean2,
fwhm2, ...]"""
    y = np.zeros_like(x)
    for i in range(0, len(params), 3):
        amp, mean, fwhm = params[i:i+3]
        y += gaussian(x, amp, mean, fwhm)
    return y

# -------------------------
# Charge-specific setup
# -------------------------
def get_charge_state_params(charge):
    if charge == 9:
        num_confs = 8
        ccs_bounds = [(1900, 2000), (2100, 2200), (2200, 2300),(2300, 2400),
(2400, 2500), (2500, 2600), (2600, 2700), (2750, 2900)]
        fwhm_bounds = [(20, 250), (2.0, 120.0), (2, 125), (2, 100), (2, 90),
(2,110), (2, 125), (2, 100)]
    elif charge == 10:
        num_confs = 7
        ccs_bounds = [(1700, 2150),(2250, 2400),(2400, 2500),(2500, 2600),(2700,
2800),(2800, 2900),(2950, 3100)]
        fwhm_bounds = [(2, 200.0),(2, 150.0),(2, 125),(2, 100),(2, 175),(2,
100),(2, 125)]
    else:
        raise ValueError(f"No bounds defined for charge {charge}")
    return num_confs, ccs_bounds, fwhm_bounds

# -------------------------
# Main
```

```
# --------------------------
if __name__ == "__main__":
    parser = argparse.ArgumentParser(description="Fit multiple Gaussian
conformations simultaneously.")
    parser.add_argument("--charge", type=int, required=True, help="Charge state
(e.g., 7 for 7+)")
    parser.add_argument("--input", type=str, default="input.txt", help="Two-
column input file (CCS intensity)")
    args = parser.parse_args()

    charge = args.charge
    infile = args.input

    # Load data
    data = np.loadtxt(infile)
    x = data[:, 0]
    y = data[:, 1]
    order = np.argsort(x)
    x, y = x[order], y[order]
    ysigma=np.sqrt(y)
    # Get setup
    num_confs, ccs_bounds, fwhm_bounds = get_charge_state_params(charge)

    # Build initial guesses and bounds for all Gaussians
    p0, lower_bounds, upper_bounds = [], [], []
    amp_guess = max(y)
    for i in range(num_confs):
        mean_guess = np.mean(ccs_bounds[i])
        fwhm_guess = np.mean(fwhm_bounds[i])
        p0.extend([amp_guess, mean_guess, fwhm_guess])
        lower_bounds.extend([0, ccs_bounds[i][0], fwhm_bounds[i][0]])
        upper_bounds.extend([np.inf, ccs_bounds[i][1], fwhm_bounds[i][1]])

    # Global fit
    popt, _ = curve_fit(
        multi_gaussian, x, y,
        p0=p0,
        bounds=(lower_bounds, upper_bounds),
        maxfev=50000
    )

    # Output results
    with open("output.txt", "w") as f:
        f.write(f"Charge State: {charge}+\n")
        f.write("Conf\tAmplitude\tCCS_mean\tFWHM\tArea\n")
```

```
    for i in range(num_confs):
        amp, mean, fwhm = popt[3*i:3*i+3]
        sigma = fwhm / 2.354820045
        area = amp * sigma * np.sqrt(2 * np.pi)
        f.write(f"{i+1}\t{amp:.6g}\t{mean:.4f}\t{fwhm:.4f}\t{area:.6g}\n")

# Plot results
xfit = np.linspace(x[0], x[-1], 2000)
yfit_total = multi_gaussian(xfit, *popt)

plt.figure(figsize=(8, 5))
plt.plot(x, y, 'ok', ms=2, label="Raw Data")
plt.plot(xfit, yfit_total, 'k', lw=2, label="Total Fit")
for i in range(num_confs):
    ycomp = gaussian(xfit, *popt[3*i:3*i+3])
    plt.plot(xfit, ycomp, lw=1.5,linestyle='--', label=f"Conf {i+1}")

plt.xlabel("CCS")
plt.ylabel("Intensity")
plt.title(f"Charge {charge}+ Multi-Gaussian Fit")
plt.legend()
plt.tight_layout()
plt.xlim(1500,3500)
plt.savefig("CCS_fit.png", dpi=300)
plt.close()
print("Results written to output.txt")
```
